# Humans program artificial delegates to accurately solve collective-risk dilemmas but lack precision

**DOI:** 10.1073/pnas.2319942121

**Published:** 2025-06-16

**Authors:** Inês Terrucha, Elias Fernández Domingos, Rémi Suchon, Francisco C. Santos, Pieter Simoens, Tom Lenaerts

**Affiliations:** ^a^Internet technology and Data Science lab, Department of Information Technology, Ghent University-IMEC, Ghent 9052, Belgium; ^b^Artificial Intelligence lab, Computer Science Department, Vrije Universiteit Brussel, Brussels 1050, Belgium; ^c^Machine Learning Group, Département d’Informatique, Université Libre de Bruxelles, Brussels 1050, Belgium; ^d^FARI Institute, Université Libre de Bruxelles-Vrije Universiteit Brussel, Brussels 1050, Belgium; ^e^Anthropo Lab - Experience, Transhumanism, Human Interactions, Care & Society, Maison des Chercheurs, Université Catholique de Lille, Lille 59000, France; ^f^Instituto de Engenharia de Sistemas e Computadores: Investigação e Desenvolvimento em Lisboa and Instituto Superior Técnico, Universidade de Lisboa, Porto Salvo 2744-016, Portugal; ^g^Center for Human-Compatible Artificial Intelligence, UC Berkeley, Berkeley, CA 94702

**Keywords:** delegation, autonomous agents, behavioral experiments, collective-risk dilemma, surprise restart

## Abstract

In an era increasingly influenced by autonomous machines, it is only a matter of time before strategic individual decisions that impact collective goods will also be made virtually through the use of artificial delegates. Through a series of behavioral experiments that combine delegation to autonomous agents and different choice architectures, we pinpoint what may get lost in translation when humans delegate to algorithms. We focus on the collective-risk dilemma, a game where participants must decide whether or not to contribute to a public good, where the latter must reach a target in order for them to keep their personal endowments. To test the effect of delegation beyond its functionality as a commitment device, participants are asked to play the game a second time, with the same group, where they are given the chance to reprogram their agents. As our main result we find that, when the action space is constrained, people who delegate contribute more to the public good, even if they have experienced more failure and inequality than people who do not delegate. However, they are not more successful. Failing to reach the target, after getting close to it, can be attributed to precision errors in the agent’s algorithm that cannot be corrected amid the game. Thus, with the digitization and subsequent limitation of our interactions, artificial delegates appear to be a solution to help preserving public goods over many iterations of risky situations. But actual success can only be achieved if humans learn to adjust their agents’ algorithms.

## Significance

Even though delegation to autonomous agents might affect the outcome of our decision-making, many questions pertaining to when and how delegation provides a positive or a negative impact remain unexplored. We are specifically interested in how the individual delegation of strategic decisions affects situations wherein individuals must consider short-term personal costs to secure future collective gains. Our results show that people who delegate are more likely to contribute to a public good and correct previous group failure by increasing their contributions when confronted with a new instance of the same game. However, precision errors impede delegation groups from achieving efficient success. Moving forward, research into additional mechanisms is still needed to make artificial delegates a viable solution to collective problems.

In recent years, our society has been digitally transformed at an incredible pace, accommodating the growing need for virtual interactions to sustain social and economic output. While this transformation was particularly accelerated as a response to a global health crisis, which generated a need for new ways to maintain social contact while complying with the distancing measures, it has also disrupted the way we learn and work together (1, 2). Many pressing issues are now being handled virtually, and as a consequence, one can no longer imagine the process of collective decision-making—especially when there are many international stakeholders involved—without the use of a computer at the meeting table. As our society continues along this path of technological assistance, the demand for assistance by automated delegates will only increase. Indeed, the emerging bot industry—already responsible for handling the investments, logistics, and customer support for many organizations (3)—is expected to expand to many other industries, assisting and replacing humans in a variety of strategic decisions (4, 5). Artificial agents within autonomous cars (6) will be deciding on behalf of the owner which path to take, possibly impacting traffic on the public road. If electrical, it might then be dependent on an autonomous household grid manager that decides how much, and when, to burden the community’s electrical grid (7) with the charging of the vehicle. These bots, algorithms, artificial delegates, or automated proxies—all synonyms for software decision-making machines developed to carry on our goals in this digital era—hold the promise to facilitate the strategic interactions required from any business leader in the current globalized world (4, 5). However, even if these human strategists would have full control over their agent’s algorithm (and fully understand it), it is still unknown how having their decisions being made by autonomous agents would affect the collective outcome.

From human-to-human delegation literature, we learned that delegation can be used to shift blame away from the principal^*^ when selfish decisions are made (8–10). In the context of artificial delegates, barriers like the alignment problem (4), i.e. the challenge of aligning human values with machine automation without that ending in unethical or catastrophic situations, or sheer algorithmic aversion, (11–13) pose added obstacles to their deployment at scale. As a matter of course, questions related to how delegation to autonomous agents affects the outcome of strategic decision-making processes are widely addressed from a variety of disciplines, using various methodologies (14–24). On the one hand, research conducted in different contexts (19–25) suggests that both delegation to (or even sheer interaction with) autonomous machines will spur more selfish and cheating behavior among humans, just as in the principal-agent literature (8–10), where actually delegating to a machine has been found to help avoid punishment more easily (25) than delegating to another human. On the other hand, a more positive view on delegation to autonomous agents was envisioned in ref. 14, which has been supported by several experimental works (15–17). In these, delegation is found to favor fairer behavior when there are conflicts-of-interest between one’s own personal gains and the collective good. It is argued that such cooperative behavior is observed in delegation scenarios because artificial delegates serve as commitment devices (18): Artificial delegates are chosen before observing any (possibly selfish) behavior from others, with only the collective goal in mind and which cannot be changed amid the game. While that research provides important baselines, different essential questions remain unexplored in order to fully grasp the intricacies of delegation in this context. For instance, how robust are these earlier observations to changes in the strategic problem the agents address? Can artificial delegates still serve as commitment devices (18) if their principals are given the opportunity to revise their algorithms when confronted with new iterations of collective challenges? How does the freedom of choice in the agent design affect how goals are translated into the decision-making algorithm used by the artificial delegate?

To answer these questions, we designed a series of online behavioral experiments that investigate 1) how an unforeseen repetition of the collective challenge—wherein participants can revise the parameters of their agent’s algorithm—affects the group outcome once they are able to “recommit” (or not) and 2) how this effect is enhanced or diminished by having a different number of decision parameters to tweak in the agent’s algorithm (where we vary the granularity (26) of the action space). The context of this decision-making experiment is provided here by the Collective Risk-dilemma (CRD) (27–32), which allows us to measure whether delegation helps groups of individuals in preserving a public good. Specifically, the CRD is a threshold public good game in which participants risk losing all their endowments if collective contributions fall short of a given threshold (33–35)(see *Game rules* in *Materials and Methods*). The nonlinear nature of the CRD combined with the possibly varying risk attitudes among the group of individuals may pose challenges to the collective success. Nonetheless, it was shown experimentally that delegation to both humans (10, 36, 37) and artificial agents (18) may enhance the capacity to coordinate in such conflict-of-interest games. We thus hypothesize that participants in delegation conditions will therefore be higher contributors and achieve higher success rates than participants in no-delegation. Based on (26), we also expect that participants in conditions with higher granularity will contribute more often to the public account. The CRD is additionally relevant for the current study as it is played over the course of many rounds (even if the payoff is only collected at the end of the game). When performed by human participants, they can at each round revise their contributions after observing what other group members are doing. A feature that is not available for humans that delegate to autonomous agents, as the algorithm cannot be adjusted amid the game. By configuring an agent, participants are forced to commit to a strategy at the beginning of the CRD, before knowing whether the other group members will try to reach the target or take the risk. As such, the CRD provides an interesting context for delegation questions. In fact, previous work (10, 18) had already investigated whether delegation to artificial agents (18) or to human representatives (10) could increase success and contributions in the CRD. The closest work to the current research is (18), where in one of the delegation treatments the participants were asked to program (or customize) different parameters of an autonomous agent that would make each round contributions on behalf of their principal (who was unable to change the agent amid the game). In ref. 10, the game is also repeated although the participants are aware that the game will be repeated so that direct reciprocity strategies might emerge, unlike in the present research. These works on delegation extend baseline experimental research on how people solve the CRD, a game design that was first proposed in ref. 27, where the risk probability was found to greatly influence the success rates and contributions of the groups playing the game. Other than delegation, communication mechanisms such as pledges (34, 38–40) and reviews of the pledges (39) or the actions of others (40) have been experimentally studied to understand whether or not they would increase success in this type of dilemma, with generally positive results (34, 38, 40). We believe that our work can be placed within the communication literature: The unforeseen repetition of the dilemma makes the first game a binding pledge and an honest signal of intentions, and the two different granularity treatments can be interpreted as being more or less restrained in communicating one’s intentions. Furthermore, we frame it as a delegation to artificial agents experiment, extending previous research on this specific solution to the CRD that was first proposed by Fernández Domingos et al. (18), where the participants cannot update the program of their artificial agents for a second round of the game.

To play the game, participants are endowed with 40 “ECoins”^†^ in the beginning of the game. We set them in groups of four and informed them that in order to keep the remainder of their endowments, they had to collectively contribute 80 ECoins to the Public Account. Each group had 10 rounds to reach this target, and in every round, each individual could only contribute a discrete amount between 0 and 4 (the specific number of choices being treatment dependent). These parameters choices follow previous literature on delegation in the CRD (10, 18) with the exception of the group size (and consequently the threshold) which we decreased from 6 to 4 (also reducing the threshold from 120 to 80), which is a condition better suited for online experiments, where the risk of participant drop-out is greater. The participants were informed that if the threshold was not met, they would lose the remainder of their endowments with a probability of 50% [following previous work on communication in the CRD (38, 40)], which is referred to as the risk of the game. In the delegation condition, instead of choosing a value in each round, they were instead asked to program an “artificial agent” that would make this decision in their place, based on the other group members’ average contributions (so that it was conditional on the actions of others) and the amount accumulated in the Public Account, which allowed them to program a switch to change strategies amid the game (see *Agent Configuration* in *Materials and Methods*, as well as *SI Appendix, Instructions* and Tables S16 and S17). To tackle the question of whether people maintain their commitments to the public good when given the opportunity to reprogram their agents, our experiment encompasses two iterations of the same CRD: After participants play the CRD once (Game 1 in *Results*), they are invited to play it a second time with the same group (Game 2 in *Results*). Note that this is not a repeated CRD in the strict sense, as the second game appears after a surprise restart (41–43) to avoid the emergence of direct reciprocity strategies in Game 1. Our experiment design thus had four different treatment conditions (*Experimental Design* in *Materials and Methods*), since we varied independently the number of choices between 3 (with the action space 0, 2, 4) and 5 (with the action space 0, 1, 2, 3, 4) and whether participants were asked to delegate or not. It differs from previous work on delegation to autonomous agents (18) in the group size (and subsequently the threshold, as it is a function of the group size), as well as in the risk probability value. We also used a risk of 50% instead of 90%. When the risk is 90% the problem is reduced to coordinating effort to solve the dilemma in order to maximize expected payoffs. Instead, using a risk of 50% allows us to understand whether some form of communication is indeed established: Are they unable to reach the target because they fail to coordinate or do they agree from the beginning (or observation of honest signaling in Game 1) to risk the collective loss? Both carry the same expected payoffs, making the 50% a more interesting risk probability to study communication and commitment.

In this manuscript, we show that when the action space is constrained, groups who delegate contribute more to the Public Account than those in the no-delegation condition. In general, we find that delegation increases the frequency of nonzero contributions to the Public Account, in any game, and independently of how many choices they are given. These results are surprising, especially since participants in the delegation treatment are also found to endure more inequality and failure in their first game-play experience. The number of choices available to choose from in each round is found to mostly affect the outcome of people who are not delegating. With higher granularity in the action space, people are able to be selfish without necessarily contributing zero, and this allows them to avoid spite from their group members and sustain high levels of cooperation. Facing a more constrained action space, groups playing by themselves (without delegation) that lose due to the presence of free-riders, collectively give up on the public good on the second game.

## Results

### When Action Space Is Constrained, Delegation Groups Contribute More to the Public Account.

**Fig. 1.**
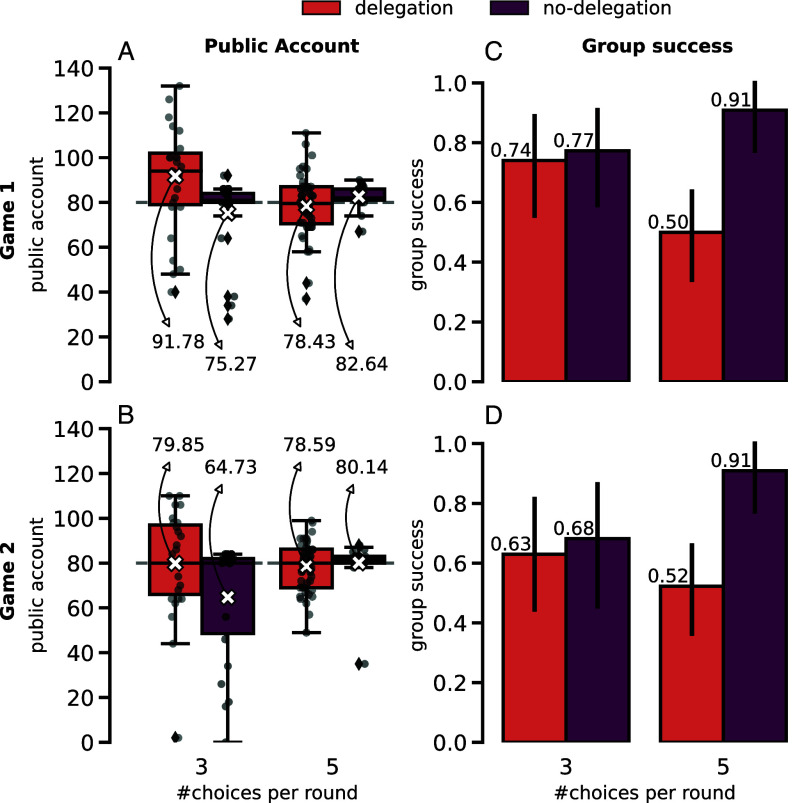
Public Account contributions and success rate observed in each of the 4 treatment conditions (total sample size of 115 groups and 460 individuals; see *SI Appendix*, Table S1) and for each of the played games (Game 1 shown in the *Top* panels and Game 2 on the *Bottom*). (*A* and *B*) present the size distributions of the Public Accounts of each group (for Game 1 and 2, respectively) across the different treatments as box plots. With a dashed line, the threshold each group had to achieve in order to keep their endowments is marked. Nonopaque points are plotted on *Top* of the box plot, representing each group value, to further illustrate the distribution of values (they are slightly dodged from each other on the x-axis to improve readability of the graph). With a white cross, the average values are marked in each box plot. The x-axis refers to the number of choices presented in that treatment to the participants. The color scheme refers to the delegation condition as is labeled in the legend on *Top* and is the same for the whole figure. Generally, delegation treatments seem to have distributions more spread out around their means (higher variation/less precision) than no-delegation. However, the no-delegation treatment is greatly skewed to negative values in the case of 3 choices in Game 2. (*C*) (Game 1) and (*D*) (Game 2) show group success across the different treatments, where the number of choices condition is shown in the x-axis as in (*A* and *B*), and the delegation/no-delegation condition is differentiated through the color scheme as identified in the legend *Above*. For both Game 1 and Game 2, we can see that there is little difference between success rates when there are 3 choices, but when there are 5 choices the no-delegation treatment appears to produce more successful groups. In all conditions, we have set group size 4, initial private endowment 40, number of rounds 10, risk 50%, and group threshold 80. When there are 3 choices, participants can choose to contribute at each round a value within the action space {0, 2, 4}; when there are 5 choices the action space is {0, 1, 2, 3, 4}.

In Fig. 1 *A* and *B*, box plots present the distribution of the amounts in the Public Accounts of the groups at the end of Game 1 (*A*) and Game 2 (*B*), and more information on the sample sizes of each treatment can be found in *SI Appendix*, Table S1. Recall that Game 2 is only played after the participants had a chance to see the outcome of Game 1 and appears to them as a “surprise” (41–43). Visual inspection of these panels shows that there appears to be a bigger variation in the delegation treatments in all conditions (than in no-delegation). In fact, delegation groups in the treatment with only 3 actions were seen to overshoot on average the threshold target in Game 1 (an average of 91.78 is found in their Public Accounts), which is corrected in Game 2 (an average of 79.85 is found for their Public Accounts, closer to the target at 80). In order to statistically compare the average Public Account values between the different treatments, we must consider the 2×2 factorial design of the experiment. Applying a two-way ANOVA test reveals a significant interaction effect in both Game 1 (*P*-value = 0.003; see *SI Appendix*, Table S10) and Game 2 (*P*-value = 0.022; see *SI Appendix*, Table S11). The presence of this interaction effect for the case of both Game 1 and Game 2 means that the combination of both variables is needed to fully explain the observed differences in the Public Accounts in Fig. 1 *A* and *B*, so that if we want to compare delegation against no-delegation, we must do that within each choice condition (i.e. 3 or 5) as the latter might affect the observed trend. Following on this, and given the great heteroskedasticity observed between the different treatment conditions’ Public Account values, we apply a one-sided Welch’s *t* test to test our hypothesis that delegation groups contribute more to the Public Account than no-delegation groups for both the 3 choices and the 5 choices treatment condition. For the 3 choices action space case, we find indeed that delegation groups contributed significantly more to the Public Account than no-delegation ones in both Game 1 (One-sided Welch *t* test: t-statistic = 2.62, *P*-value = 0.006) and Game 2 (One-sided Welch *t* test: t-statistic = 2.04, *P*-value = 0.024). However, when there are 5 choices in the action space, no significant difference was found in either Game 1 or Game 2.

In Fig. 1, we also show the success rate of the groups for Game 1 (*C*) and Game 2 (*D*). The highest success rates are observed in the treatment condition with 5 actions and no-delegation (91% in both Game 1 and Game 2; see *SI Appendix*, Tables S2 and S3). Indeed, for the case of 5 actions, groups are significantly less successful in delegation than in no-delegation treatments in Game 1 (*P*-value = 0.001, Fisher’s exact test with the corresponding contingency tables in *SI Appendix*) and Game 2 (*P*-value = 0.001, Fisher’s exact test with the corresponding contingency tables in *SI Appendix*). However, no statistical difference is found for the case of 3 actions. While there seems to be a drop in success based on the number of action choices between the delegation treatments (and an increase between the no-delegation treatments), no statistically significant effect is found, neither in Game 1 nor in Game 2.

### Individuals Who Delegate Contribute More Often to the Public Good, Despite Experiencing More In-Group Inequality.

**Fig. 2.**
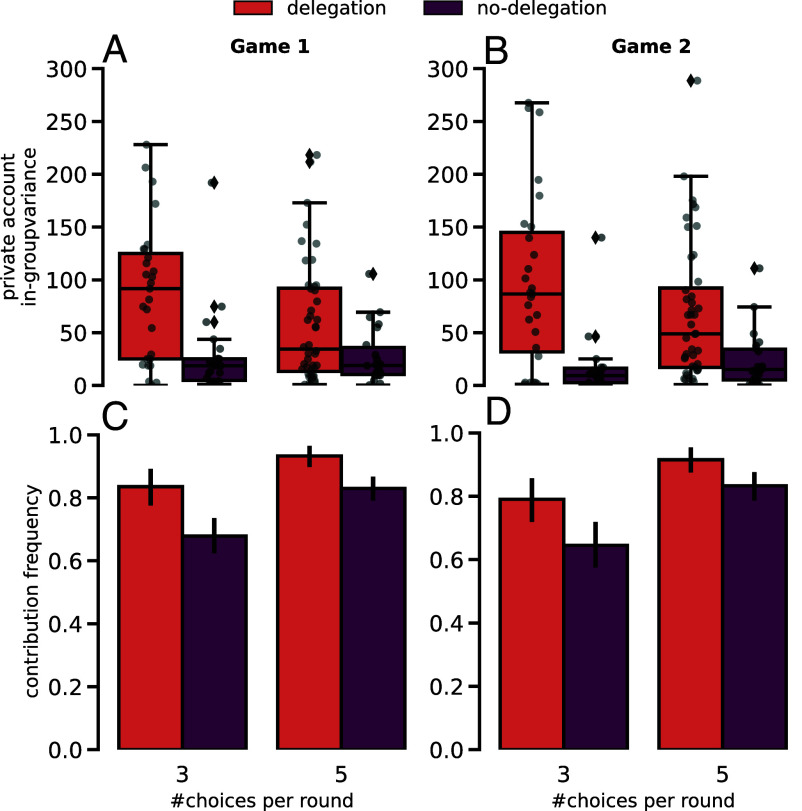
Private account within-group variance and contribution frequency (frequency of nonzero contributions) observed in each of the 4 treatment conditions (total sample size of *n* = 115 groups and N=4×115=460 individuals)—and for each of the played games (Game 1 shown in the *Left* panels and Game 2 on the *Right*). (*A*) (Game 1) and (*B*) (Game 2) plot the distribution of each group’s variance within the Private Accounts of each individual within that group, a group metric intended to identify within group asymmetries in contribution efforts. In other words, a measure of the inequality experienced by the individuals of each group. With a white cross, the average values are marked in each box plot. (*C* and *D*) display the average contribution frequency, i.e. the frequency of nonzero contributions made by each individual throughout the 10 rounds of the game, for each treatment condition, again each respecting observations made in Game 1 and Game 2.

Fig. 2 *A* and *B* show the variance of each group’s distribution of (its members’) Private Accounts for both Game 1 (*A*) and Game 2 (*B*). A two-way ANOVA shows no significance in the interaction effect between delegation and number of choices (see *SI Appendix*, Table S12 for Game 1 and *SI Appendix*, Table S13 for Game 2). The same test reveals no significant effect on the number of choices. However, in-group variances of Private Account values are higher in the delegation treatment (in both Game 1: *P*-value ≪ 0.001, and in Game 2: *P*-value ≪ 0.001) when compared with the no-delegation. In other words, there is more in-group inequality in the delegation experiments, independently of how many choices the participants are given in their action space.

Finally, Fig. 2 *C* and *D* show the contribution frequency, i.e. the frequency of nonzero contributions of each individual during the game (26) (a participant who always contributes something has a contribution frequency of 10/10 = 1). A factorial ANOVA is used to compare the contribution frequencies across the different treatment conditions (see *SI Appendix*, Table S14 for Game 1 and *SI Appendix*, Table S15 for Game 2), now considering the sample size of the total participant pool (460), given that an individual characteristic is explored here. Both delegation and a higher number of actions are found to have a significant positive effect on the average contribution frequency obtained in both Game 1 and Game 2 (*P*-value ≪0.001 in both cases), while no significant interaction effect is observed. The positive effect that higher granularity of the action space has on the contribution frequency confirms previous work (26). Because no interaction effect is found, delegation is expected to increase the contribution frequency regardless of the granularity of the action space.

### Early-Contributors Are Mainly Present When There Is No Delegation, and Therefore No Commitment.

**Fig. 3.**
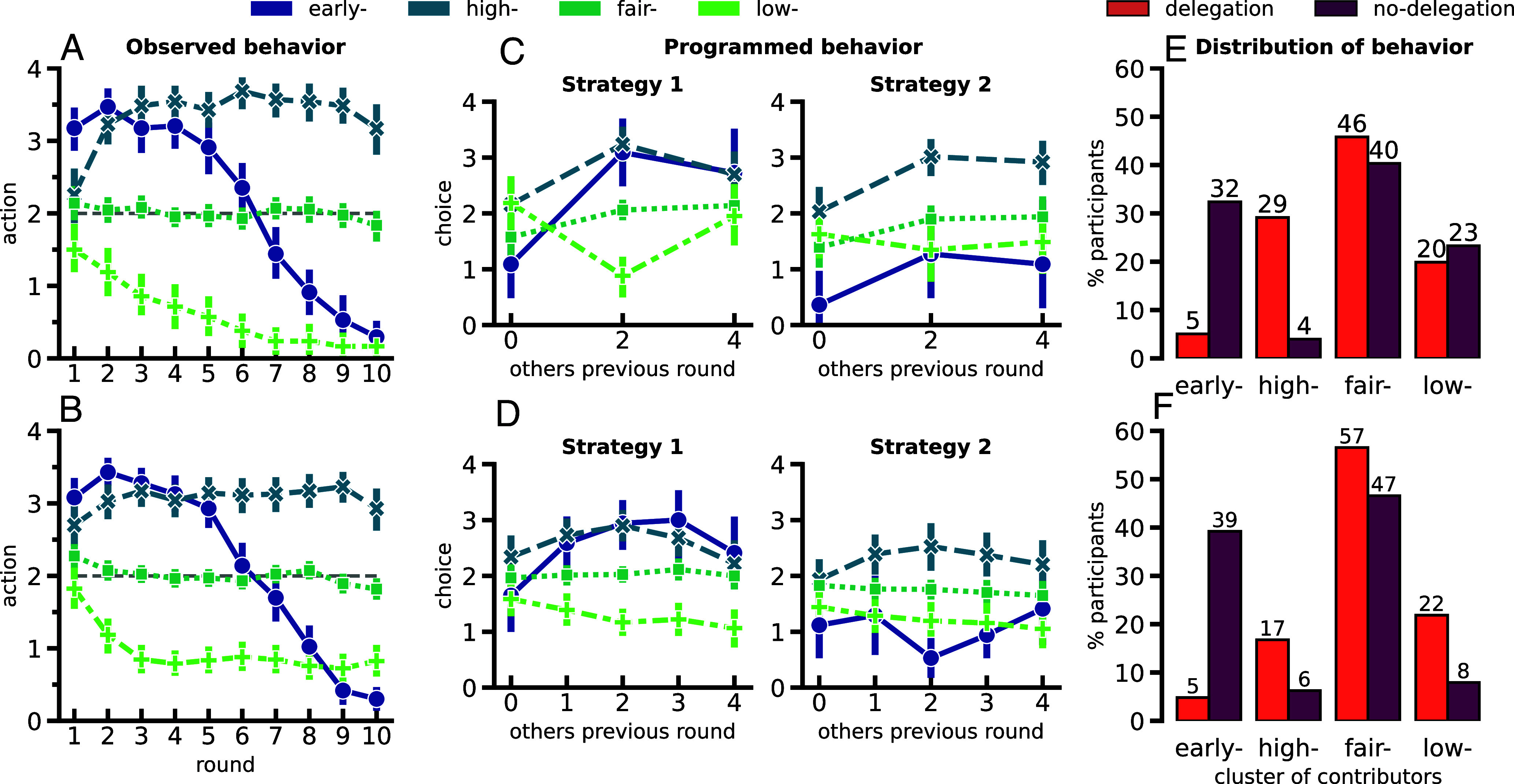
Classification of the observed individual behavior. Applying a K-Means clustering technique on the 920 10-round-by-round action vectors (460 present from each game) leads to the identification of four different behavioral profiles—the early, high, fair, and low contributors—as labeled on the *Top* legend of the figure. The *Top* row refers to the treatment conditions where there are 3 choices in the action space and the *Bottom* row to the treatment conditions with 5 choices. In (*A* and *B*), a round-by-round visualization of the average behavior observed in each cluster during the course of the game is shown. The case where 3 action choices is presented in (*A*) (392 samples), and the case of 5 action choices in (*B*) (528 samples). (*C* and *D*) show the average settings programmed—in delegation treatments only—by the participants whose behavioral profile was identified with each of the clusters labeled on the *Top* legend for both the case where 3 actions are made available [*Top* panel (*C*) with a total sample of 216] and when 5 actions are possible [*Bottom* panel (*D*) a total sample of 352]. In other words, (*C* and *D*) show which actions the participants belonging to each cluster chose (y-label is “choice”), on average, to contribute in case their group members contributed a rounded of average 0, 2, or 4 for the 3 action case (*C*) or 0, 1, 2, 3, or 4 for the 5 action case (*D*) in the previous round (x-axis “others previous round”). On the *Left* we show how they programmed their Strategy 1 (the strategy they start with) and on the *Right* their Strategy 2 (the strategy to which they switch if the Public Account reaches their personally defined switch value). For further details on the agent’s configuration, see *Materials and Methods* and *SI Appendix*, Tables S16 and S17. (*E* and *F*) show how the identified behavioral profiles are distributed within each delegation condition denoted by the color scheme on the *Top*-*Right* legend. (*E*) corresponds to the distribution within each delegation/no-delegation condition when there are 3 actions and (*F*) for when there are 5 actions. In all figures Game 1 and Game 2 behaviors are plotted together, as they were clustered together following the assumption that even though people might change behavior between games, they would follow the same heuristics.

The results illustrated in Figs. 1 and 2 show significant differences in game results for groups belonging to different treatment conditions. A study on the observed individual behavior is therefore due, to better understand how the distribution of individual behavior per treatment condition might influence the overall group results. First, the individual observed behaviors must be grouped in simple categories to facilitate their analysis. For this matter, a K-Means clustering technique (*SI Appendix, K-Means Clustering*) is performed using as input the 10-element action sequences either produced directly by the participant or indirectly by their agent. This representational choice allows for an easy comparison between delegation and no-delegation experiments. By means of the “elbow method,” the optimal number of clusters is found to be *k* = 4 (*SI Appendix*, Figs. S1–S3). Fig. 3 provides information on the main results. The panels in the *Top* row of the figure collect the results for the setting when there were 3 actions to choose from and the *Bottom* row contains the same information for the setting with 5 actions.

In Fig. 3 *A* and *B*, the average action value played in each round within the different clusters is presented, grouping together the delegation and no-delegation treatments. In both panels, early-contributors, i.e. a behavioral profile of contributions higher than 2 in early rounds and lower than 2 in later rounds of the game, can be identified. Next to this observed behavior, high-, fair-, and low-contributors are detected, representing the clusters that exhibit relatively fixed behaviors throughout the course of the game where participants contribute over all the 10 rounds higher, equal, and lower than 2, respectively. We acknowledge that both the fair-contributors and the early-contributors make game-level fair contributions of their endowments to the public good, i.e. if everyone would contribute their fair share (half of their endowments), then the target would be met and everyone would secure the same payoffs (30). However, the fair-contributors achieve this by also being fair locally: By selecting the action that provides half of what could be given in each round, no compensation by others is needed in any round.

While in the no-delegation treatment participants acted themselves in each round, by choosing how much to contribute to the Public Account in every round, in the delegation experiment they were asked to program an agent to do so for them. In this sense, the (average) actions shown in Fig. 3 *A* and *B* were not a direct choice of the delegation participants, but rather a consequence of how they programmed their agents to act and with whom they were grouped with—had they been in a different group, they could have acted differently. In fact, even though most observed behaviors in the delegation treatment are identified as high-, fair-, and low-contributors—all with (a somewhat) fixed round-by-round behavior-, most participants programmed delegates whose contributions depend on others’ contributions. Only 17 individuals in the delegation treatment with 5 choices (9.7% of the 176 total) and 15 in the delegation treatment with 3 choices (13.9% of the 108 total) have actually chosen the exact same action for the different parameters that make up Strategy 1; of which only 7 in the delegation treatment with 5 choices (4% of the 176 total) and 9 in the delegation treatment with 3 choices (8.3% of the 108 total) also chose the same parameters for Strategy 2 and have therefore programmed a truly fixed-behavior strategy on their agents. The mapping between observed and programmed behavior is mainly established when we consider the middle range of values of “others previous round.” As Fig. 3 *C* and *D* hint visually, some of the more extreme values, for example, when “others previous round” is 0 or 4, would actually motivate different actions from the agents than the ones the behavior they were clustered in. For this reason, Fig. 3 *C* and *D* show the actually programmed behavior that participants in the delegation treatments have configured their agents with, where we can see that regardless of how fixed the behavior of the agents appears during the game, the majority of them were programmed to have conditional behavior. These *Middle* panels are divided into two plots: one dedicated to their action choices for Strategy 1 and one for the ones of Strategy 2 (see *Agent Configuration* in *Materials and Methods* and consult the *SI Appendix, Instructions* given to the participants in *SI Appendix* for more details on how the agents were programmed). Both Strategy 1 and Strategy 2 plots tell us how participants chose, on average, for their agents to respond to others’ behavior in the previous round.

What is interesting is that one can now analyze how the round-by-round play results (*A* and *B*) relate to the choices made by the participants when programming their agents’ settings (*C* and *D*). Indeed, upon performing a statistical analysis on the middle parameters (when others contribute 1, 2, or 3 in the previous round) of Fig. 3 *C* and *D*, we are able to establish a comparison between the programmed conditional settings and the observed behavior in round-by-round play. We observe that while early- and high- contributors are not distinguishable from one another in Strategy 1, both clusters appear as significantly higher contributors than fair- or low-contributors, whereas fair- still contribute higher than low-contributors. Instead, while in Strategy 2 the relationships established between high-, fair-, and low- are maintained, early-contributors now appear to contribute lower than high- and fair-contributors together with the low-contributors. A Welch’s *t* test was used to conduct the detailed analysis for the comparison between the clusters settings within the different number of choices conditions (see *SI Appendix*, Table S9 for details on the hypothesis tested, the t-statistic, and the *P*-values obtained).

To understand how these behaviors are distributed within each delegation vs. no-delegation treatment, we turn to Fig. 3 *E* and *F*, where again we use the *Top* panel *E* for the 3 actions treatments and the *Bottom* panel *F* for the 5 actions. Importantly, two salient discrepancies are found between delegation and no-delegation conditions, independently of how many actions were made available to them (3 or 5): A bigger fraction of participants behaves as high-contributors in delegation treatments than in no-delegation (Fisher’s exact test: *P*-value ≪0.001; see *SI Appendix*, Table S4), while early-contributors represent a larger portion of behaviors when participants play by themselves than when they have to delegate (Fisher’s exact test: *P*-value ≪0.001; see *SI Appendix*, Table S5). These findings are consistent with Fig. 1, where delegation groups are more likely to overshoot the target. Overall, one can also observe that the fair-contributors strategy is the most prevalent independently of the treatment condition participants were allocated to. In *E*, we see that it takes 46% and 57% of the delegation and no-delegation total sample of individual behavior for the 3 action treatment. For the 5 action treatment, we show in *F* that similarly it takes 40% and 47% of the total no-delegation and delegation treatments. When comparing treatments with different action spaces, and consistent with what is presented in Fig. 2 *C* and *D*, low-contributors represent a larger fraction of behaviors in the case of 3 actions than in the case of 5 actions, a difference that is only significant in no-delegation treatments (Fisher’s exact test: *P*-value ≪0.001; see *SI Appendix*, Table S6).

### Failure in Game 1 Only Significantly Lowers Average Contributions and Success Probability in Game 2 for No-Delegation.

**Fig. 4.**
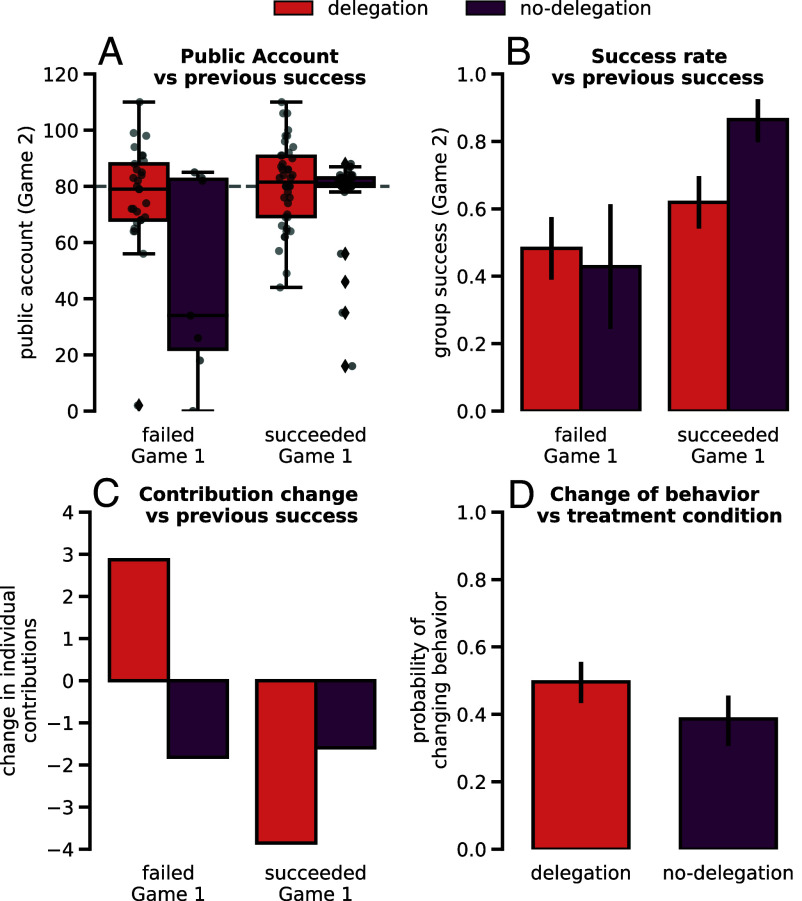
How Game 1 influences Game 2, and who made the most changes between games. (*A*) shows the distribution of Public Account values in Game 2 given its delegation condition and whether the group was successful or failed in Game 1 through the use of box plots. Colors indicate whether values pertain to delegation or no-delegation treatment and follow the legend on *Top* of the figure (the same colors will differentiate between delegation and no-delegation through the rest of the figure). Nonopaque points represent the samples corresponding to the values of each group and their deviance on the x-axis has no meaning and is only meant to aid the visualization of different samples with the same value. With a white cross, the average values are marked in each box plot. (*B*) uses bar-plots to present success rate of groups in Game 2 given their previous success in Game 1 and whether the group belongs to a delegation or a no-delegation treatment. (*C*) focuses on how individuals change their contributions between Game 1 and Game 2 (Game 2 contributions minus Game 1 contributions) given their success in Game 1 and whether they were assigned to a delegation or a no-delegation treatment. (*D*) shows the probability of an individual changing their allocated cluster of behavior (as identified in Fig. 3) between Game 1 and Game 2 given their delegation treatment. A total of 42 groups succeeded Game 1 in the delegation treatment (29 failed), and 37 succeeded Game 1 in the no-delegation treatment (7 failed).

While Figs. 1–3 reveal how groups fare and how individuals act in the different treatment conditions of Game 1 and Game 2, they do not reveal yet how behavior is adapted between games. Fig. 4 provides now these insights focusing particularly on how delegation compares to no-delegation treatments.

Fig. 4 *A* and *B* illustrate how group success and Public Account group contributions could be dependent on previous collective success obtained by each group between delegation treatments. In Fig. 4*A*, we observe no significant difference when comparing the distribution of Public Account values between previously failed and previously successful groups in the delegation treatment. In contrast, previous success seems to play a role in group contributions to the Public account in the no-delegation treatments. In this case, groups who have previously failed greatly skew their group contributions to even lower values when they are asked to play a second time when compared to groups who succeeded (Welch’s *t* test: t-statistic = 2.22, *P*-value = 0.03, for the hypothesis that Public Account values in Game 2 are greater when groups experienced previous success in Game 1 in the no-delegation treatment). In Fig. 4*B*, we again see no significant link between previous and future collective success obtained by groups within the delegation treatment. This again contrasts with the no-delegation case, where previous success does have a positive effect on future success (Fischer’s exact test: *P*-value = 0.024; see *SI Appendix*, Table S7): Success in the second game is more likely if the group was already successful in the previous game.

Looking now at individual changes in behavior, panel *C* shows the difference in contributions made by each individual between Game 2 and Game 1 (where a positive value stands for an increase in contributions between games and a negative value represents a decrease). On the one hand, we observe no statistical difference between the change in contributions made by an individual of the no-delegation treatment based on their previous success. On the other hand, in the delegation treatment, we confirm the hypothesis that individuals who were in a failed group greatly increase their contributions between games in comparison with individuals who belong to a group that succeeded in the first game (Welch’s *t* test: t-statistic = 5.12, *P*-value ≪ 0.001). This trend pointing toward the fact that individuals are more likely to adapt their behavior when they are in a delegation treatment is further supported by Fig. 4*D*. Indeed, applying a Fisher’s exact test to the data in panel *D* shows that delegation participants are significantly more likely to change their behaviors than no-delegation participants (*P*-value = 0.013; see *SI Appendix*, Table S8).

## Discussion

When looking at Fig. 1 *A* and *B* to try and pinpoint the effect of delegation within the context of the CRD, we get immediately reminded of any other diagram used to explain the difference between accuracy and precision (44) (see *SI Appendix*, Fig. S5 for an illustration). Independently of the number of choices available to participants, we can see that no-delegation groups were much more precise in reaching the goal, with a very low variance around the dashed line that illustrates the target 80, than those who had to delegate their decisions and whose Public Account values are more spread out around the average. Focusing on treatments with 3 actions, another interesting effect can be noted: While in Game 1 delegation groups also had low accuracy, overshooting the target with an average Public Account bigger than 90, in Game 2 they are able to correct their accuracy, bringing it much closer to 80. In contrast, no-delegation groups with 3 actions seem to lose their accuracy from Game 1 to Game 2, which appears with a distribution greatly skewed toward values lower than 80 (so perhaps with the intention to not even try to reach the collective target). The overshooting of delegation groups in Game 1 and the negative skewness of no-delegation groups in Game 2 are sufficient to make Public Account contributions in delegation groups significantly greater than the contributions made by no-delegation groups when there are only 3 actions. Interestingly, the same negative skewness is not observed in the no-delegation treatment with 5 choices. In that case, we hypothesize that because the more selfish participants are able to be so without necessarily contributing nothing [by contributing 1 at least; see also Fig. 2 and (26)], the other group members might feel less provoked, and are then more likely to keep on contributing despite the presence of lower (but nonzero) contributors in the group. However, due to their low precision, even though delegation groups contribute either similarly or more to the Public Account, they do not achieve higher success rates (Fig. 1 *C* and *D*). Even when the average contribution is almost superpositioned with the target, around half of the groups are still short of meeting it, leading to success rates around 50% (5 choices condition). Nonetheless, those who delegate show improvement in accuracy in their second game by keeping their aim on the target (rather than quitting on it, as it appears to happen in no-delegation groups during the second game when there are only 3 choices).

This effect is especially interesting if we take into consideration that delegation groups experience more in-group inequality (Fig. 2 *A* and *B*), i.e. some group members end up contributing much more/less than others, when compared with individuals in no-delegation groups, where we see that individuals belonging to the same group make similar contributions. One would expect that such inequality would trigger feelings of betrayal between group members (45), leading to negative adjustments between games. Indeed, failure to coordinate in achieving the collective target in no-delegation groups is often attributed to such feelings, who are known to corrupt cooperation efforts once a group member identifies another as a free-rider and stops contributing themselves (18). On the contrary, delegation individuals increase their contributions after experiencing failure in the first game (Fig. 4*C*). They also contribute more often to the Public Account than no-delegation groups as is shown by their contribution rates in Fig. 2 *C* and *D*, apparently undeterred by previous failures in the first game. The question then becomes: Do people become immune to betrayal feelings when they delegate because they understand selfish behavior as a mistake rather than a strategy? or do people who delegate simply prioritize the collective task at hand—avoiding the collective risk—over reacting or punishing other cogroup members’ behavior? Another interpretation could be that in the delegation treatment, since participants make their decisions in advance, they are in a “cold” mental state, instead of reacting to the actions of others in a “hot” headed state (46, 47). This may lead them to rely more on deliberation, and less on emotions in the delegation treatment. One thing is clear, if attitudes toward adjustment of behavior propagate through many iterations of the same collective dilemma, even though delegation groups do not start with higher success rates (due to lack of precision to the target), delegation seems fundamental to sustain cooperation in the long term.

It is worth noting that we have obtained a surprisingly high success rate for the treatment condition with 5 actions and no-delegation, much higher than its delegation counterpart, thus contrasting with previous observations (18). The differing results may be a consequence of various factors. First, in ref. 18 groups of size 6 were used instead of 4. Although on the one hand, bigger group sizes make coordination more difficult (48), on the other hand, delegation may produce a more positive effect, since agents respond to the contributions made by a group rather than looking at each of the five other individuals independently. Delegation acts then as a more powerful correlation device. Second, the action set of 3 actions increases the frequency of zero-contributions throughout the game, impeding sustained cooperation through the various rounds of the game. This echoes previous work on the effect of the granularity of action space on group success (26). Finally, the risk used in ref. 18 was 90% rather than 50%, which might have spurred more emotional responses from the participants when confronted with selfish behavior from their peers. We acknowledge that other works have probed the CRD with a risk of 50% with no delegation (27, 40), where (40) also uses a smaller group size of three individuals. However, both these experimental works frame the game to the participants as a climate change problem, which might have influenced their contributions in unforeseen ways (no study on the participants’ attitudes toward this topic is disclosed in either work). This makes it difficult to establish a direct comparison between our work and theirs. Nonetheless, the high variance in the Private Account between group members and in the Public Account is consistently reported both in this work and in ref. 18.

Even though aggregate group results (Fig. 1) have already proven to be quite enlightening about the effect of delegation in coordinating in a CRD, the contrasting in-group inequality observed in Fig. 2 *A* and *B* points us to look into the distribution of individual behavior within each treatment condition. Following on this, we identify four different behavioral profiles in how individuals contribute at each round of the game, shown in Fig. 3*A* (3 actions) and *B* (5 actions), which we name as the early-, the high-, the fair-, and the low-contributors. It is interesting to see how even though the average contribution is almost fixed throughout the 10 rounds of play for the delegation case (Fig. 3 *E* and *F*), very few participants actually programmed their agents with the same values for every case (Fig. 3 *C* and *D*). Even though participants program their agents with conditional actions on how to respond to other people’s behavior, the resulting course of action taken by their agents in a given group will be almost fixed throughout the game. It is possible that some agents will end up compensating the lack of contributions of others, keeping a constant average group contribution that propagates what appears to be a fixed individual behavior. Such would also explain the high in-group inequality observed in Fig. 2 *A* and *B*.

Furthermore, Fig. 3 *E* and *F* tell us that in contrast with the no-delegation treatment, where early contributors seem to share the space mainly with fair contributors, early-contributors rarely appear in the delegation conditions. Moreover, we see that there are barely any high contributors in the no-delegation treatments, while these together with the low-contributors make up the second big part of the delegation treatment agents—possibly a result of participants in the no-delegation treatment being able to adjust their contributions constantly along the game. As already mentioned, delegation participants find themselves “stuck” with a certain course of behavior until the end of the game-play, resulting in the observed poor precision when trying to reach the target as a group. Again, we confirm the effect of granularity in contribution frequency, especially in the case of no-delegation where we see that people avoid contributing nothing in the 5 choices treatment: There are much fewer low-contributors in the 5 choices treatment than in the 3 choices one.

The emergence of early contributors as one of the main behaviors within our no-delegation treatments also appears as a surprise given what had been previously studied in the literature: Earlier (modeling) work specifically led to opposing conclusions (28). However, the model developed in that work assumed that the game would end after reaching the target. If this assumption is dropped, the model returns similar distributions for both fair and early contributors, as is observed in the no-delegation treatment results shown in this manuscript. Experimental work led by the authors of refs. 18 and 30 also favored procrastinating behavior rather than early contributions, although as previously mentioned, the group size in that work was 6 rather than 4. Group size may influence participants’ trust in their group members to coordinate on reaching the goal, an effect already probed in refs. 48 and 49. A lack of trust may thereafter lead them to procrastinate their collective efforts until they realize that every other group member is also keen on collaborating toward it.

Finally, one of the main contributions of this work is centered on how people change their behavior between games. Apart from refs. 10 and 50, our work is one of the few providing an experimental perspective on repeated CRD games. In our work, the second game comes as a surprise to the participants after having completed the first. Our goal was to deter them from using direct reciprocity in the first game, so that we would be able to identify how they adapt their behavior once their beliefs were updated about how to play the game with their current group members. In other words, with the increased experience about how others play this game, how do people change their behavior? Strikingly, Fig. 4 further elaborates on what we had already hinted toward while analyzing Fig. 1. People who do not delegate appear as quite sensitive on previous group outcomes when deciding whether or not to cooperate to reach the target, while those who do delegate exhibit similar group behavior, whether they have been successful or not in the past. In Fig. 4*A*, we see how participants from the no-delegation treatment that have previously failed contribute very little on average to the Public Account on the second game, once again hinting toward some sentiments of grudge or betrayal (45). Interestingly, looking at Fig. 4 *C* and *D* where the focus is on how individuals, rather than groups, change their behavior we see that even though the group outcome is visibly more different with regard to the previous outcome in the no-delegation case, it is those who delegate that make most changes (*D*). Participants in the no-delegation treatment on average decrease their contributions, whether they had been successful or not. Contrarily, participants in the delegation treatment appear to try to correct previous precision mistakes: If they were successful they contribute less on the second game, whereas if they failed, they contribute more (recall Fig. 1*A* where it is clearly shown how most of them either over- or undershoot the target in Game 1).

Fig. 4 therefore contributes further to support that group failure in the delegation treatment only happens as a fruit of mistake, rather than intention. Such contrasts with the no-delegation treatment, where the previous outcome appears to strongly dictate whether or not they will be able to succeed in future endeavors with the same group members. It is interesting that even when given the opportunity to reprogram their agents and tackle a new problem together, participants in the delegation treatment seem untouched by feelings of betrayal and are therefore able to keep their heads “cold” on the target (46). One hypothesis to be explored in the future is that players are less likely to punish other players who acted as low-contributors in the game, if they are able to see how they programmed their agents. As in Fig. 3, most agents are programmed with conditional behavior, and upon seeing that low-contributors are not free-riders by design, other players might approach their second game together with a forgiving (rather than punishing) attitude.

In this paper, we have conducted a thorough analysis on how delegation can constitute a solution to sustain cooperation through consecutive CRDs, even when the risk is low. Not only do people who delegate contribute more often to the public good in each game, but they are also more likely to correct previous failures when confronted with a new game by contributing more on the second. Even though they might miss the group target due to precision errors, they aim for the target and appear to not become spiteful toward their cogroup members after having experienced in-group inequality. This resilience effect, rather than a coincidence, appears to be a trait to be associated with delegated decision-making. It summarizes the glaring disparity observed in individual and group behavior between delegation and no-delegation treatments, where in the latter, people are seen to give up early on the group target perhaps to punish low-contributors, even if their group was previously on track for success. The contrast between the two granularity conditions (3 choices vs. 5 choices) in the human-play conditions also points out that humans can be extremely prosocial when the space of strategies is big enough to allow some form of signaling to emerge (51), but their cooperative skills are highly impaired once that space is shrunk and messages of selfish behavior echo louder, consequently hurting future hopes of success. In a world where an increasing number of social interactions become digital, and our capacity to communicate with a wider array of behavioral actions (through gestures, facial expressions, or different tones of voice) is debilitated, our work shows that delegation can be a way to keep collective goals in mind. When it comes to achieving efficient success in overcoming collective success with artificial agents, the precision errors identified in this manuscript point toward a learning process ahead. However, delegation does seem to enable the creation of a collective mind, that withstands in-group inequality and keeps individuals focusing on how to better achieve collective success out of a risky situation.

## Materials and Methods

### Experimental Design.

Our experiments follow a 2×2 (Number of choices × (No-)Delegation) factorial design: We had participants play the CRD game with 3 (S=0,2,4) or 5 (S=0,1,2,3,4) different options on how much to contribute at each round, with and without delegating their decisions to an autonomous agent set up by themselves (see *SI Appendix*, Table S1 to consult the 4 treatment conditions used throughout the paper and respect sample sizes). We had participants play a first CRD (Game 1) followed by a surprise restart (41–43) where we asked them to play a second CRD (Game 2). The surprise restart was performed similarly to what was done in ref. 43, in order to avoid the effect of direct reciprocity on the strategies conceived by the participants to play the first CRD game (see the full *SI Appendix, Instructions* for further detail). Between games, participants were able to see how each group member fared in Game 1 (amount earned and success of the group). Participants in the delegation treatment were also able to observe how each other had programmed their agents for Game 1. Before the participants engaged in the actual game-play part of the experiment, they were asked to complete a small questionnaire on their attitudes toward the use of automated decision-making systems. They were also subject to a comprehension test on the Instructions presented to them to assert their suitability to participate in it and to ensure that they have understood the task fully. A postexperiment questionnaire was presented to them in the end, aimed at qualitatively assessing their game preferences, expectations, and overall satisfaction on their experience. This experiment was designed to be conducted online, as is evident in *SI Appendix, Instructions and Questionnaires*. Participants were recruited online through Prolific (52) where they gained access to our platform, designed by means of the lioness lab tool (53). Informed consent was obtained from all participants (*SI Appendix, Invitation Letter and Informed Consent*). The research was approved by the Ethics committee of the Faculty of Psychology and Educational Sciences of Ghent University. All data and code used to produce the results reported within this manuscript are available at https://doi.org/10.5281/zenodo.10805220 (54).

### Game Rules.

The CRD is a threshold public goods game, meaning that it is played between more than two players and the game payoffs are not linearly mapped to each player’s contributions to the public good. Instead, a group of players must reach a collective threshold of contributions to a public good to unlock their own personal rewards. In the case of the CRD, the participants start the game with an initial endowment from which they make contributions to the public good, and their rewards correspond to the remaining of this endowment once the collective threshold is met. It is also a game with delayed reward, i.e. the players are expected to make small contributions over a finite number of rounds but are only able to collect a reward at the end of the game. What makes this a risk dilemma is the fact that if the players are unable to meet the threshold, their loss is not certain but rather probabilistic. At the end of the game, if the group is not successful, its individuals only lose their endowments in accordance to a risk factor, known to them at the start of the game. Due to the fact that it can be used to abstract the collective dilemma we face when trying to tackle climate change and other prominent global issues, it has attracted a lot of attention in recent years. The effect of having different risk factors, group sizes, endowments, thresholds, or number of rounds have been studied in different experimental (27, 30, 38) and theoretical works (28, 31, 55, 56). Here, we fix the risk to 50%, the number of rounds to 10, the group size to 4, the initial endowment to 40 and the threshold to 80. To test the behavioral impact of having different action spaces, we devise two choice conditions: one where 3 different actions are allowed in each round of the game (0, 2, 4) and one where 5 different actions are possible (0, 1, 2, 3, 4).

### Agent Configuration.

In our delegation treatment, we ask participants to “configure an artificial agent” that will play the game on their behalf instead of choosing an action at every round. In order to configure an agent, participants have to fill in a table that will fully determine the choices made by their algorithm in each round depending on what others have done in the previous round and on how much is currently held in the public account. The settings within this table can be divided in four parts: i) the starting action, ii) a first strategy for the agent to follow, iii) a switch to change strategies, and iv) a second strategy. The starting action is the action their agent plays in the first round. The parts corresponding to the agent’s algorithmic strategies were inspired by a simplified version of the strategy method^‡^ (47, 57, 58) already presented in previous public good experiments (45, 59). Equivalently, the participants in this experiment will also have to decide how much to contribute if the others in their group have contributed, on average and rounded to the closest element, each of the values also accessible to them in their respective action space^§^. The first part of this configuration table is then dedicated to “Strategy 1” or how the agents start playing the game after the starting action is played (on round 2, agents start acting conditionally on how other players have chosen their starting actions). In our experiment, we allow participants to switch strategies amid the game, although they have to preprogram this switch before the game is played, by using the same configuration table. To switch between strategies, the participants must choose a value between 0 and 120: Whenever the public account reaches this value chosen by them, their agent will stop following the first strategy to determine its course of action and will instead start following the second strategy. The agent’s second strategy, “Strategy 2,” follows the same logic as the first, as is previously depicted. In each of the decisions made in either the starting action or the strategy parts of the table, the participants will have to choose between elements made available to them in their assigned strategy space, may it be (0, 2, 4) or (0, 1, 2, 3, 4). In both “Strategy 1” and “Strategy 2,” the participants are then only able to react to how others played in the previous round. With the addition of a switch and the possibility to craft a “Strategy 2” that is different from “Strategy 1,” we also enable them to be strategic about the amount accumulated in the public account, or in other words, goal-oriented. We provide every participant recruited to take part in this study with thorough instructions of how to configure their artificial agents and we test their knowledge before doing so, so that we can ensure that every participant truly knows how to configure their agent to accommodate their best interests when playing the presented game on their behalf. Both the instructions, the test, and the configuration table can be consulted in *SI Appendix*.

## Supplementary Material

Appendix 01 (PDF)

Dataset S01 (CSV)

Dataset S02 (CSV)

Dataset S03 (CSV)

Dataset S04 (CSV)

Dataset S05 (CSV)

## Data Availability

Anonymized .csv data have been deposited in zenodo DOI: https://doi.org/10.5281/zenodo.11033297 (54).

## Footnotes

^*^From the principal-agent literature on human-to-human delegation, the principal is the one who delegates a task to an agent.

^†^Participants are fully informed that their ECoins will be exchanged to Pounds (£) at the end of the experiment at an exchange rate of 1ECoin = 0.1£.

^‡^The strategy method would require the participants to define a plan of action for all possible sets of beliefs. To remain usable and therefore useful, delegation devices necessarily restrict the number of parameters one can set. Even though institutions, policies, or contract designs could have a similar effect, the agents in this case are programmed directly and solely by their users, conveying therefore only their personal preferences, though constrained by the set of parameters available to them.

^§^For example, when their action space in each round corresponds to the set (0, 1, 2, 3, 4), they will have to make 5 decisions: how much to contribute if the others in their group contributed 0, 1, 2, 3, or 4, respectively. If they are participating in an experimental treatment where the action space is (0, 2, 4) then they only have to make 3 decisions: how much to contribute if the others have on average and rounded to the closest element, contributed 0, 2, or 4.
