# Supplementary material for: Humans program artificial delegates to accurately solve collective-risk dilemmas but lack precision
Source: Proc Natl Acad Sci U S A. 2025 Jun 16;122(25):e2319942121. doi: 10.1073/pnas.2319942121 (PMC12207457; doi:10.1073/pnas.2319942121)
Supplement: Supplementary file 1 — Appendix 01 (PDF) [file pnas.2319942121.sapp.pdf]

Supporting Information for

Humans program artificial delegates to accurately solve collective-risk dilemmas but lack precision

Inês Terrucha, Elias Fernández Domingos, Rémi Suchon, Francisco C. Santos, Pieter Simoens and Tom Lenaerts

Tom Lenaerts.
E-mail: Tom.Lenaerts@ulb.be

This PDF file includes:

Supporting text
Figs. S1 to S5
Tables S1 to S18
Legends for Dataset S1 to S5
SI References

Other supporting materials for this manuscript include the following:

Datasets S1 to S5

Supporting Information Text

Statistical tests

Within this section of the Supplementary Information material we elaborate on the reasoning that led us to choose the statistical tests we used in the main manuscript to assert the significance of our main results. For the case of the Fisher's exact test we also add the contingency tables needed to reproduce our results. For the case of the two-way ANOVA test, we add the summary table of results. In all cases we explain how the test was implemented, namely the python package used to aid us in the process, as well as its online resources.

Fisher's exact test. A Fisher's exact test is used to assess the significance of the results as shown through contingency tables. Contingency tables are tables that display the frequency distribution of the variables. Within the main text of the manuscript we rely on the Fisher's exact test to test the significance of the effect that the treatment condition variables have on binary outcomes such as: the success rate in Fig.1 of the main manuscript (see Tables S2 and S3), the frequency of each individual behavior in Fig.3 of the main manuscript (see Tables S5, S4 and S6), the frequency of cluster changes in Fig.4 (see Table S8) and the frequency of successful groups given previous success in the same Fig.4 (see Table S7). The python package *scipy.stats.fisher_exact* (https://docs.scipy.org/doc/scipy/reference/generated/scipy.stats.fisher_exact.html#scipy-stats-fisher-exact) was used to compute the p-values associated with this test in the main text. This function takes as input a 2×2 contingency table and the specification of the alternative hypothesis, which can be: 'two-sided': the odds ratio of the underlying population is not one; 'less': the odds ratio of the underlying population is less than one; or 'greater': the odds ratio of the underlying population is greater than one.

Welch's t-test. The Welch's t-test, sometimes called unequal variances t-test, is used to test whether two samples have equal means (two-sided test) or whether one sample's mean is greater/smaller than the other (one-sided) without assuming equal variances (or even equal sample sizes). Given how it generalizes the student's t-test (by dropping the equal variance assumption), it is the test we rely on throughout the manuscript to compare means of distributions. Namely, this test is used to analyze the means of the Public Account values (Fig. 1); when comparing action and settings values between clusters in Fig. 3 (see Table S9 for more detail on the analysis of Fig. 3); and when comparing Public Account values given previous success or failure in Fig. 4A. The python package *scipy.stats.ttest_ind* (https://docs.scipy.org/doc/scipy/reference/generated/scipy.stats.ttest_ind.html) was used to compute the p-values associated with this test in the main text. This function takes as input two arrays corresponding to the two samples meant to be compare, the specification of the alternative hypothesis ('two-sided': the means of the distributions underlying the samples are unequal; 'less': the mean of the distribution underlying the first sample is less than the mean of the distribution underlying the second sample; 'greater': the mean of the distribution underlying the first sample is greater than the mean of the distribution underlying the second sample) and a parameter "equal_var" which we turn False to perform the test without assuming equal variance between the populations. The p-values obtained are specified within the main text, as well as the alternative hypothesis used.

Two-way ANOVA. The two-way ANOVA is an extension to the one-way ANOVA that permits the assessment of the effect of two categorical independent variables on one continuous dependent variable. As a result of this test, we are able to determine if either of the categorical independent variables has an effect on the continuous dependent one, but also if the interaction between the two independent variables also has an effect on the continuous variable. For this reason, we find this test especially suited to assess the significance of our variables of interest within our factorial 2×2 experimental design. Specifically, we apply this test on the continuous variables shown in Figs. 1 and 2 of the main text: for Public Account see Tables S10 and S11, for in-group Private Account variance see Tables S12 and S13, for Participation Frequency see Tables S14 and S15. To implement this test, we use the python package *pingouin.anova* (https://pingouin-stats.org/generated/pingouin.anova.html), which takes as input the dataset we are working on (the package is based on pandas), the name of the column that represents the dependent variable, and the names of the independent variables as a list in the input "between". As a result from this test, we retrieve a summary table of the ANOVA. Below we present the resulting tables for the results present in the main text.

Invitation letter and informed consent

In this section we present the text that first appeared to the participants once logged in our experimental platform. In order to participate in the experiment, they were required to read and accept this informed consent letter. In this informed consent the participants were able to learn about who is responsible for the study, what is the purpose of the study, what kind of data would be recorded, how much they would be able to earn and if there were any risks or discomfort feelings they should know about. To consent, we asked them to write their Prolific IDs in order for the button "Continue to the experiment" to appear.

INVITATION LETTER AND INFORMED CONSENT

(Your consent is needed in order for you to be able to participate in our experiment.)

INVITATION LETTER

This letter gives you information about the scientific study we are conducting, in order for you to make an informed decision about whether or not you consent hereto and wish to participate in it. We encourage you to read carefully. If you still have some questions, please message the experimenters in the Prolific messaging system. You can also ask questions after the experiment is finished through the same messaging system, namely you can ask for a summary of the results once the study is

finished and published. If you decide to participate, you will be asked to sign this form (by ticking a box in the Informed Consent displayed below) and stay online until the end of the experiment, which will be made clear to you with the display of a Prolific completion code. However, if you do not feel comfortable you can leave before the experiment ends.

Who organizes this study:

The experiment is conducted by researchers at the Ghent University and the Vrije Universiteit Brussel. These two universities are the controllers of the data and will both be receiving the data in order to use and retain it for the purposes of scientific research. All the experiments of this group are done in accordance with the General Data Protection Regulation (GDPR - The General Data Protection Regulation 2016/679 is a regulation in EU law on data protection and privacy in the European Union and the European Economic Area.) and follow the practice and protocols traditionally followed in experimental economics. As a 'data subject' the GDPR grants you several rights that you can exercise over your personal data: for more information you can contact the data protection officers of the UGent (privacy@ugent.be) or the VUB (dpo@vub.be). If you feel your personal data has been handled incorrectly, you can always contact the supervisory authority, the Belgian Data Protection Authority (https://www.dataprotectionauthority.be/) at contact@apd-gba.be.

What is purpose of the study:

This experiment studies how individuals make decisions and its purpose is therefore to get to know more about this. Through a computer interface, you will be asked to make decisions within the context of strategic games, involving other participants. In this manner, you will be interacting in a group with 3 other real people who also accepted this task, who are completing it at the same time. Therefore, it is important that you complete this task without interruptions. The choices you make in the experiment will directly contribute to the amount of money you earn. Further instructions about the specific rules of the game and how the decisions you and the rest of your group make influence your final payment will be provided to you in the instructions section that directly follows this general information letter. We do not expect any particular behavior from you. Deception is strictly forbidden in the protocols of the type of economic experiments we perform here. There is thus no deception in this experiment and all the information needed to perform this experiment is given to you in the experimental instructions.

What kind of data will be recorded:

During the experiments, even though you will be interacting with 3 other real human participants, your identity or even your Prolific ID will never be shown to other participants. You and all other participants will be identified through pseudonyms (for example, through the letters A, B, C and D) for the purpose of the interaction during the strategic games. Nor you, nor the other participants will ever know with whom they have interacted in this experiment. The experimenter will use your Prolific ID to confirm your payment after reviewing the submissions, and will afterwards discard it from the experimental collected data. Your actions in the game will never be linked to your Prolific ID, since the Prolific ID will be discarded from the dataset once your submission has been confirmed. Only the actions you take in the game will be stored and made available to the scientific community (indefinitely, since they accompany and corroborate the publication of the results), as well as summary statistics of the demographics of the whole subject pool (which at this point will not be traceable to you as an individual, not even through your Prolific ID, which will be discarded before – and therefore does not constitute any personal data).

How long it will last and how much will you earn:

The experiment will last around 30 min and the amount you earn is determined by a completion fee of 2.5£ plus your earnings in the strategic games that can go up to 8£ more. In total, you can earn up to 10.5£, where 2.5£ will be credited to you automatically upon completion of this task and the rest, corresponding to your earning within the game, will be credited to you through the bonus payments option, as soon as possible, within 5 working days. If you do not get matched with 3 other participants and are therefore unable to complete the experiment, you are still entitled to the completion fee of 2.5£ (as long as you do not leave abruptly before the completion code for this task appears in your screen).

Is there a risk for your health or a discomfort you might feel?

There are no health risks and the risk of the discomfort is very small. Most participants really enjoy the experience; however, you might get disappointed with the amount of money you earned and/or other people's behavior. Please, let us know if you need to talk to us after the experiment through the Prolific messaging system.

Pre-Experiment Questionnaire

The following questions were asked to all participants regardless of the experimental treatment to which they were assigned.

Q1. Do you know what an algorithm or automated decision-making (ADM) system is? *[answer: Yes/No/Not sure]*

Q1a. *[if Q1=Yes]* Could you tell us how you understand algorithms and ADM systems? (optional) *[answer: open text]*

Q1a. *[if Q1=Not sure]* Could you try and tell us what you think it might be? (optional) *[answer: open text]*

Q2. Would you let an algorithm/ADM system make an important decision instead of you in one of the following areas?

Area 1: Social media (news feed and content recommendations) *[answer: Yes/No/Not sure]*

Area 2: News (news recommendations) *[answer: Yes/No/Not sure]*

Area 3: Entertainment (recommendation of movies, songs, videos etc) *[answer: Yes/No/Not sure]*

Area 4: Transport (individual or public, transport of goods) *[answer: Yes/No/Not sure]*

Area 5: Home (heating, energy, lights) *[answer: Yes/No/Not sure]*

Area 6: Finance (loans, investing) *[answer: Yes/No/Not sure]*

Area 7: Healthcare (diagnosis, therapy, medicine, cure) *[answer: Yes/No/Not sure]*

Q2.1 For the questions you answered to with "Yes", could you briefly elaborate why would you let an algorithm decide instead of you? (optional) *[answer: open text]*

Q2.2 For the questions you answered to with "No", could you briefly elaborate why would you not let an algorithm decide instead of you? (optional) *[answer: open text]*

Q2.3 For the questions you answered to with "Not sure", could you briefly elaborate why you were not sure? (optional) *[answer: open text]*

Instructions

In this section of the Supplementary Information we present a transcript of the Instructions presented to the participants in each different experimental treatment. Please refer to Tables S16 and S17 in order to understand how the agent's configuration table was presented to the participants in the delegation treatment. When asked to program an agent, they were required to specify each of the values in the table that are shown on the left side in all caps. This representation of the agent's strategy was inspired by the version of the strategy method (1–3) already presented in other public goods experiments (4, 5) that were conducted without a delegation motivation.

Delegation with 3 choices. Welcome to this experiment!

You are about to participate in an experiment on decision-making, conducted by researchers from the Vrije Universiteit Brussel and the Ghent University, and where you will earn real money. The amount you make will depend on your choices and the choices of the other participants in your group (more information will follow).

Please do not close this window or leave the task's web pages in any other way during the task. If you close your browser or leave the task abruptly, you will not be able to re-enter, and we will not be able to pay you! Furthermore, you will be spoiling the experience of the other 3 players in your group - also human and recruited through Prolific - and with whom you will be interacting synchronously (more information will also follow).

Including the time for reading these instructions, the task will take about 30 minutes to complete. To complete this task, you will be asked to:

- Read Instructions (and complete a comprehension test on them);
- Participate in Decision-Making games with other participants;
- Complete a Survey on your experience;

These parts may contain more subsections, which may or may not be different. In any case, you will always be informed about what to expect in each part - these tasks involve NO deception.

All your earnings from the decision-making part will be expressed in Experimental Coins (ECoins), which will be transformed into Pounds with a change rate of 1 ECoin = 0.1 £. You will earn a completion fee of 2.5£ for your time and attention during this task. If by chance you cannot be matched with 3 other participants, you are still entitled that completion fee of 2.5£ for your time (unless you leave abruptly before you arrive at the screen where your completion code is shown). If there is any problem in the course of this tasks or after, please contact the experimenters through the Prolific messaging system.

Throughout this experiment, your privacy is guaranteed: the other participants will never be able to identify you during or after the experiment and the results of the experiment will be stored anonymously.

Instructions - What are the rules of the game?

To play the Decision-Making games, you will be matched to 3 other participants to form a group of 4 participants in total.

You will only interact with those 3 other group members - also human and recruited through Prolific - throughout the course of this task.

Every member of the group will receive the same instructions, and will have the same decisions to make.

Below we explain the rules of the game, and how both your decision and the decisions of the other group members will influence your earnings in this task.

Once you have been matched with 3 other participants, you and everyone in your group will receive a personal endowment of 40 ECoins, which will be credited to your private account.

A public account will also be created, and will start with 0 ECoins.

A game consists of 10 rounds. Before round 1, you must configure an artificial agent that will act on your behalf during the experiment, according to a procedure that will be described to you in Instructions - How do you configure the artificial agent? on the next screen (after you correctly answer the comprehension test below).

At each round, the artificial agent will contribute 0, 2 or 4 ECoins from your private account to the public account of the group.

You will be able to observe the actions that your agent and the other agents took at each round, but you will not be able to change your agent's behavior (once you have set up your agent for the game, you cannot change its behavior or start playing by yourself manually).

At every round, you will also be able to observe the content of the public account and the content of your private account (where the remainder of your endowment is stored).

After the 10 rounds,

- If the Public Account reaches 80 ECoins, then you keep the ECoins left in your Private Account. These will be exchanged for Pounds £ at an exchange rate of 1 ECoin = 0.1 £.
- If the Public Account does not reach 80 ECoins, then a virtual coin is tossed (50%/50% chance, like a coin flip): if it is "heads" you still get to keep the ECoins left in your Private Account, but if it is "tails", you lose all the Ecoins you had left.

Note also that the coin flip is the same for everyone in your group: either all of you get "heads" and keep the remainder, or all of you get "tails" and lose the remainder of your Private Accounts.

Instructions - How to configure your artificial agent? (see also Table S16)

As described before, instead of playing the game yourself, you will configure an artificial agent. In practice, you will basically plan the actions your agent should take in the next round, by considering the actions of the other group members made in each current round as well as the current amount in the Public Account.

To configure your artificial agent, you will have to:

1. Decide how much to contribute in the starting round (START);
2. Fill 2 tables - TABLE 1 and TABLE 2 - which will determine the action your agent takes in the following round, by considering the decisions made in the current;
3. In each table, for each given ROW X (X can be 0, 2 or 4), you must decide how much your agent should contribute in the next round (0, 2 or 4) if the other members of your group gave X on average (where average is rounded to the closest number - 0, 2 or 4 -, without decimals - *see note before comprehension test) in the current round;
4. You can decide on different values for the contributions your agent should give in each table;
5. Your agent will first follow TABLE 1, until the Public Account reaches a value determined by you (a whole-number, without decimals, determined by you, between 0 and 160), after which, in the following round, your agent will follow TABLE 2 until the end of the game - this will be represented by the parameter SWITCH in the configuration table of the agent;
6. In total you will have to decide on 8 different parameters to configure your agent: START, TABLE 1 ROW 0, TABLE 1 ROW 2, TABLE 1 ROW 4, SWITCH, TABLE 2 ROW 0, TABLE 2 ROW 2 and TABLE 2 ROW 4.

After passing the comprehension test below and after being matched with the 3 other participants, you will be asked to configure your agent for the game.

Then, before your decision is final, you will be able to observe a table with the full configuration of your agent that looks like the example below* (where instead of "START", "TABLE X ROW Y" and "SWITCH" you will see the numeric input corresponding to the decisions you made).

You will be asked to confirm the configuration of the agent before moving forward to the game.

Once you confirm the configuration of your agent, you will not be able to change it. Recall that the configuration of your agent will influence your final earnings of this task.

Then, you will be able to observe the decisions made by all the agents in your group in every round as well as your final results from the game.

*Note on how to calculate the average contribution of the other 3 players rounded to the nearest integer:

- AVERAGE: You have to sum the contributions of all the other group members - except yourself - and divide by 3 (since they are 3).
- ROUND TO THE CLOSEST NUMBER 0, 2 OR 4: 2/3 is 0.667 which is closer to 0 (than 2 or 4). 4/3 is 1.333 which is closer to 2 (than 0 or 4). 8/3 is 2.667 which is also closer to 2 (than 0 or 4). 10/3 is 3.333 which is closer to 4 (than 0 or 2).

Delegation with 5 choices. Welcome to this experiment!

You are about to participate in an experiment on decision-making, conducted by researchers from the Vrije Universiteit Brussel and the Ghent University, and where you will earn real money. The amount you make will depend on your choices and the choices of the other participants in your group (more information will follow).

Please do not close this window or leave the task's web pages in any other way during the task. If you close your browser or leave the task abruptly, you will not be able to re-enter, and we will not be able to pay you! Furthermore, you will be spoiling the experience of the other 3 players in your group - also human and recruited through Prolific - and with whom you will be interacting synchronously (more information will also follow).

Including the time for reading these instructions, the task will take about 30 minutes to complete. To complete this task, you will be asked to:

- Read Instructions (and complete a comprehension test on them);

*In this Supplementary Information, instead of below, please check Table S16.

- Participate in Decision-Making games with other participants;
- Complete a Survey on your experience;

These parts may contain more subsections, which may or may not be different. In any case, you will always be informed about what to expect in each part - these tasks involve NO deception.

All your earnings from the decision-making part will be expressed in Experimental Coins (ECoins), which will be transformed into Pounds with a change rate of 1 ECoin = 0.1 £. You will earn a completion fee of 2.5£ for your time and attention during this task. If by chance you cannot be matched with 3 other participants, you are still entitled that completion fee of 2.5£ for your time (unless you leave abruptly before you arrive at the screen where your completion code is shown). If there is any problem in the course of this tasks or after, please contact the experimenters through the Prolific messaging system.

Throughout this experiment, your privacy is guaranteed: the other participants will never be able to identify you during or after the experiment and the results of the experiment will be stored anonymously.

Instructions - What are the rules of the game?

To play the Decision-Making games, you will be matched to 3 other participants to form a group of 4 participants in total.

You will only interact with those 3 other group members - also human and recruited through Prolific - throughout the course of this task.

Every member of the group will receive the same instructions, and will have the same decisions to make.

Below we explain the rules of the game, and how both your decision and the decisions of the other group members will influence your earnings in this task.

Once you have been matched with 3 other participants, you and everyone in your group will receive a personal endowment of 40 ECoins, which will be credited to your private account.

A public account will also be created, and will start with 0 ECoins.

A game consists of 10 rounds. Before round 1, you must configure an artificial agent that will act on your behalf during the experiment, according to a procedure that will be described to you in Instructions - How do you configure the artificial agent? on the next screen (after you correctly answer the comprehension test below).

At each round, the artificial agent will contribute 0, 1, 2, 3 or 4 ECoins from your private account to the public account of the group.

You will be able to observe the actions that your agent and the other agents took at each round, but you will not be able to change your agent's behavior (once you have set up your agent for the game, you cannot change its behavior or start playing by yourself manually).

At every round, you will also be able to observe the content of the public account and the content of your private account (where the remainder of your endowment is stored).

After the 10 rounds,

- If the Public Account reaches 80 ECoins, then you keep the ECoins left in your Private Account. These will be exchanged for Pounds £ at an exchange rate of 1 ECoin = 0.1 £.
- If the Public Account does not reach 80 ECoins, then a virtual coin is tossed (50%/50% chance, like a coin flip): if it is "heads" you still get to keep the ECoins left in your Private Account, but if it is "tails", you lose all the Ecoins you had left.

Note also that the coin flip is the same for everyone in your group: either all of you get "heads" and keep the remainder, or all of you get "tails" and lose the remainder of your Private Accounts.

Instructions - How to configure your artificial agent? (see also Table S17)

As described before, instead of playing the game yourself, you will configure an artificial agent. In practice, you will basically plan the actions your agent should take in the next round, by considering the actions of the other group members made in each current round as well as the current amount in the Public Account.

To configure your artificial agent, you will have to:

1. Decide how much to contribute in the starting round (START);
2. Fill 2 tables - TABLE 1 and TABLE 2 - which will determine the action your agent takes in the following round, by considering the decisions made in the current;
3. In each table, for each given ROW X (X can be 0, 1, 2, 3 or 4), you must decide how much your agent should contribute in the next round (0, 1, 2, 3 or 4) if the other members of your group gave X on average (where average is rounded to a whole-number, without decimals - *see note before comprehension test) in the current round;
4. You can decide on different values for the contributions your agent should give in each table;
5. Your agent will first follow TABLE 1, until the Public Account reaches a value determined by you (a whole-number, without decimals, determined by you, between 0 and 160), after which, in the following round, your agent will follow TABLE 2 until the end of the game - this will be represented by the parameter SWITCH in the configuration table of the agent;

6. In total you will have to decide on 12 different parameters to configure your agent: START, TABLE 1 ROW 0, TABLE 1 ROW 1, TABLE 1 ROW 2, TABLE 1 ROW 3, TABLE 1 ROW 4, SWITCH, TABLE 2 ROW 0, TABLE 2 ROW 1, TABLE 2 ROW 2, TABLE 2 ROW 3 and TABLE 2 ROW 4.

After passing the comprehension test below and after being matched with the 3 other participants, you will be asked to configure your agent for the game.

Then, before your decision is final, you will be able to observe a table with the full configuration of your agent that looks like the example below[†] (where instead of "START", "TABLE X ROW Y" and "SWITCH" you will see the numeric input corresponding to the decisions you made).

You will be asked to confirm the configuration of the agent before moving forward to the game.

Once you confirm the configuration of your agent, you will not be able to change it. Recall that the configuration of your agent will influence your final earnings of this task.

Then, you will be able to observe the decisions made by all the agents in your group in every round as well as your final results from the game.

*Note on how to calculate the average contribution of the other 3 players rounded to the nearest integer:

- AVERAGE: You have to sum the contributions of all the other group members - except yourself - and divide by 3 (since they are 3).

- ROUND TO A WHOLE-NUMBER: Dividing by 3 will give you numbers with decimals .33, .66 or without any decimals. If the decimals end in .33 you consider the previous number (if 2.33, then you consider 2); if the decimals are .66 you consider the next whole-number (if 2.66, then you consider 3).

No-Delegation with 3 choices. Welcome to this experiment!

You are about to participate in an experiment on decision-making, conducted by researchers from the Vrije Universiteit Brussel and the Ghent University, and where you will earn real money. The amount you make will depend on your choices and the choices of the other participants in your group (more information will follow).

Please do not close this window or leave the task's web pages in any other way during the task. If you close your browser or leave the task abruptly, you will not be able to re-enter, and we will not be able to pay you! Furthermore, you will be spoiling the experience of the other 3 players in your group - also human and recruited through Prolific - and with whom you will be interacting synchronously (more information will also follow).

Including the time for reading these instructions, the task will take about $totalTime$ minutes to complete. To complete this task, you will be asked to:

- Read Instructions (and complete a comprehension test on them);

- Participate in Decision-Making games with other participants;

- Complete a Survey on your experience;

These parts may contain more subsections, which may or may not be different. In any case, you will always be informed about what to expect in each part - these tasks involve NO deception.

All your earnings from the decision-making part will be expressed in Experimental Coins (ECoins), which will be transformed into Pounds with a change rate of 1 ECoin = 0.1 £. You will earn a completion fee of 2.5£ for your time and attention during this task. If by chance you cannot be matched with 3 other participants, you are still entitled that completion fee of 2.5£ for your time (unless you leave abruptly before you arrive at the screen where your completion code is shown). If there is any problem in the course of this tasks or after, please contact the experimenters through the Prolific messaging system.

Throughout this experiment, your privacy is guaranteed: the other participants will never be able to identify you during or after the experiment and the results of the experiment will be stored anonymously.

Instructions - What are the rules of the game?

To play the Decision-Making games, you will be matched to 3 other participants to form a group of 4 participants in total.

You will only interact with those 3 other group members - also human and recruited through Prolific - throughout the course of this task.

Every member of the group will receive the same instructions, and will have the same decisions to make.

Below we explain the rules of the game, and how both your decision and the decisions of the other group members will influence your earnings in this task.

Once you have been matched with 3 other participants, you and everyone in your group will receive a personal endowment of 40 ECoins, which will be credited to your private account.

A public account will also be created, and will start with 0 ECoins.

A game consists of 10 rounds. At each round, you will choose whether to contribute 0, 2 or 4 ECoins from your private account to the public account of the group.

You will be able to observe the actions that you and every other member of your group take at each round.

[†] In this Supplementary Information, instead of below, please check Table S17.

At every round, you will also be able to observe the content of the public account and the content of your private account (where the remainder of your endowment is stored).

After the 10 rounds,

- If the Public Account reaches 80 ECoins, then you keep the ECoins left in your Private Account. These will be exchanged for Pounds £ at an exchange rate of 1 ECoin = 0.1 £.

- If the Public Account does not reach 80 ECoins, then a virtual coin is tossed (50%/50% chance, like a coin flip): if it is "heads" you still get to keep the ECoins left in your Private Account, but if it is "tails", you lose all the Ecoins you had left.

Note also that the coin flip is the same for everyone in your group: either all of you get "heads" and keep the remainder, or all of you get "tails" and lose the remainder of your Private Accounts.

No-Delegation with 5 choices. Welcome to this experiment!

You are about to participate in an experiment on decision-making, conducted by researchers from the Vrije Universiteit Brussel and the Ghent University, and where you will earn real money. The amount you make will depend on your choices and the choices of the other participants in your group (more information will follow).

Please do not close this window or leave the task's web pages in any other way during the task. If you close your browser or leave the task abruptly, you will not be able to re-enter, and we will not be able to pay you! Furthermore, you will be spoiling the experience of the other 3 players in your group - also human and recruited through Prolific - and with whom you will be interacting synchronously (more information will also follow).

Including the time for reading these instructions, the task will take about 30 minutes to complete. To complete this task, you will be asked to:

- Read Instructions (and complete a comprehension test on them);

- Participate in Decision-Making games with other participants;

- Complete a Survey on your experience;

These parts may contain more subsections, which may or may not be different. In any case, you will always be informed about what to expect in each part - these tasks involve NO deception.

All your earnings from the decision-making part will be expressed in Experimental Coins (ECoins), which will be transformed into Pounds with a change rate of 1 ECoin = 0.1 £. You will earn a completion fee of 2.5£ for your time and attention during this task. If by chance you cannot be matched with 3 other participants, you are still entitled that completion fee of 2.5£ for your time (unless you leave abruptly before you arrive at the screen where your completion code is shown). If there is any problem in the course of this tasks or after, please contact the experimenters through the Prolific messaging system.

Throughout this experiment, your privacy is guaranteed: the other participants will never be able to identify you during or after the experiment and the results of the experiment will be stored anonymously.

Instructions - What are the rules of the game?

To play the Decision-Making games, you will be matched to 3 other participants to form a group of 4 participants in total.

You will only interact with those 3 other group members - also human and recruited through Prolific - throughout the course of this task.

Every member of the group will receive the same instructions, and will have the same decisions to make.

Below we explain the rules of the game, and how both your decision and the decisions of the other group members will influence your earnings in this task.

Once you have been matched with 3 other participants, you and everyone in your group will receive a personal endowment of 40 ECoins, which will be credited to your private account.

A public account will also be created, and will start with 0 ECoins.

A game consists of 10 rounds. At each round, you will choose whether to contribute 0, 1, 2, 3 or 4 ECoins from your private account to the public account of the group.

You will be able to observe the actions that you and every other member of your group take at each round.

At every round, you will also be able to observe the content of the public account and the content of your private account (where the remainder of your endowment is stored).

After the 10 rounds,

- If the Public Account reaches 80 ECoins, then you keep the ECoins left in your Private Account. These will be exchanged for Pounds £ at an exchange rate of 1 ECoin = 0.1 £.

- If the Public Account does not reach 80 ECoins, then a virtual coin is tossed (50%/50% chance, like a coin flip): if it is "heads" you still get to keep the ECoins left in your Private Account, but if it is "tails", you lose all the Ecoins you had left.

Note also that the coin flip is the same for everyone in your group: either all of you get "heads" and keep the remainder, or all of you get "tails" and lose the remainder of your Private Accounts.

Post-Experiment Questionnaire

After the experiment, the participants that took part in the various experimental treatments are asked about their experience during the game, namely how satisfied they are with their earnings and the performance of their group in both game 1 and game 2. In this regard, some of the questions are different between delegation vs. no-delegation experimental treatments (we do not ask about their satisfaction with their agents in the no-delegation treatments, for example) and depending on whether their group was successful or not (the questions about group performance are dependent on how their group fared). Therefore, the following sub-sections indicate whether its questionnaire was shown to delegation or no-delegation participants.

Delegation. The following is the questionnaire shown at the end to all participants that took part in delegation experiments. This questionnaire is divided in 4 different parts. In the Part 1, the summary of the results of game 1 is shown again to the participants. In Part 2, the summary of both game 1 and game 2 are shown to the participants.

Part 1

1.1. Which of the following would best describe your feeling of control when configuring the agent for game 1?

1. I did not feel in control at all.
2. I did not feel in control.
3. I slightly felt not in control.
4. I felt neutral about my sense of control.
5. I felt slightly in control.
6. I felt in control.
7. I felt completely in control.

1.2. *[if 1.1. > 4]* Could you briefly tell us why you felt (at least slightly) in control? (optional) *[answer: open text]*

1.2. *[if 1.1. < 4]* Could you briefly tell us why you did not feel (even if just slightly) in control? Could you also tell us briefly what you would need to feel more in control? (optional) *[answer: open text]*

1.2. *[if 1.1. = 4]* Could you also tell us briefly what you would need to feel more in control? (optional) *[answer: open text]*

2. How satisfied were you with the performance of the agent you configured to play in game 1?

1. Completely dissatisfied
2. Mostly dissatisfied
3. Somewhat dissatisfied
4. Neither satisfied or dissatisfied
5. Somewhat satisfied
6. Mostly satisfied
7. Completely satisfied

3. How do the contributions of the agent compare with the contributions you would have made in game 1?

1. The agent contributed much less than I would.
2. The agent contributed less than I would.
3. The agent contributed slightly less than I would.
4. The agent contributed about the same as I would.
5. The agent contributed slightly more than I would.
6. The agent contributed more than I would.
7. The agent contributed a lot more than I would.

4. *[if group was successful in game 1]* How satisfied were you with your group achieving the Public Account target of 80 ECoins in game 1?

1. Completely dissatisfied
2. Mostly dissatisfied
3. Somewhat dissatisfied
4. Neither satisfied or dissatisfied
5. Somewhat satisfied
6. Mostly satisfied
7. Completely satisfied

4. *[if group was not successful in game 1]* How satisfied were you with your group NOT achieving the Public Account target of 80 ECoins in game 1?

1. Completely dissatisfied
2. Mostly dissatisfied
3. Somewhat dissatisfied
4. Neither satisfied or dissatisfied
5. Somewhat satisfied
6. Mostly satisfied
7. Completely satisfied

5. Which of the following best describe the expectations you had for the contributions of the other agents in your group in game 1?

1. The other agents contributed much less than I expected.

2. The other agents contributed less than I expected.
3. The other agents contributed slightly less than I expected.
4. The other agents contributed about the same as I expected.
5. The other agents contributed slightly more than I expected.
6. The other agents contributed more than I expected.
7. The other agents contributed a lot more than I expected.

6. *[if group was not successful in game 1 but they kept the earnings]* How satisfied were you with being able to keep the earnings of game 1, even if your group did not achieve the target?

1. Completely dissatisfied
2. Mostly dissatisfied
3. Somewhat dissatisfied
4. Neither satisfied or dissatisfied
5. Somewhat satisfied
6. Mostly satisfied
7. Completely satisfied

6. *[if group was not successful in game 1 and they didn't keep the earnings]* How satisfied were you with NOT being able to keep the earnings of game 1, after your group not achieving the target?

1. Completely dissatisfied
2. Mostly dissatisfied
3. Somewhat dissatisfied
4. Neither satisfied or dissatisfied
5. Somewhat satisfied
6. Mostly satisfied
7. Completely satisfied

Part 2

1.1. Which of the following would best describe your feeling of control when configuring your second agent (for game 2) in comparison to the feeling you had when configuring the agent for game 1?

1. I felt much less in control when configuring the second agent.
2. I felt less in control when configuring the second agent.
3. I felt slightly less in control when configuring the second agent.
4. I felt the same in control when configuring the second agent.
5. I felt slightly more in control when configuring the second agent.
6. I felt more in control when configuring the second agent.
7. I felt a lot more in control when configuring the second agent.

1.2. *[if 1.1. > 4]* Could you briefly tell us why you felt (at least slightly) more in control? (optional)

1.2. *[if 1.1. < 4]* Could you briefly tell us why you felt (even if just slightly) less in control? Could you also tell us briefly what you would need to feel more in control? (optional)

1.2. *[if 1.1. < 4]* Could you also tell us briefly what you would need to feel more in control? (optional)

2. How satisfied were you about being able to configure a new agent to play in game 2?

1. Completely dissatisfied
2. Mostly dissatisfied
3. Somewhat dissatisfied
4. Neither satisfied or dissatisfied
5. Somewhat satisfied
6. Mostly satisfied
7. Completely satisfied

3. How satisfied were you with the performance of your new second agent in game 2 in comparison to the performance of the agent you used in game 1?

1. Much more dissatisfied
2. More dissatisfied
3. Slightly more dissatisfied
4. About the same
5. Slightly more satisfied
6. More satisfied
7. Much more satisfied

4. *[if group was successful in game 2]* How satisfied were you with your group achieving the Public Account target of 80 ECoins in game 2?

1. Completely dissatisfied
2. Mostly dissatisfied
3. Somewhat dissatisfied
4. Neither satisfied or dissatisfied

5. Somewhat satisfied
6. Mostly satisfied
7. Completely satisfied

4. *[if group was not successful in game 2]* How satisfied were you with your group NOT achieving the Public Account target of 80 ECoins in game 2?

1. Completely dissatisfied
2. Mostly dissatisfied
3. Somewhat dissatisfied
4. Neither satisfied or dissatisfied
5. Somewhat satisfied
6. Mostly satisfied
7. Completely satisfied

5. Which of the following best describe the expectations you had for the contributions of the other agents in your group in game 2?

1 - The other agents contributed much less than I expected.
2 - The other agents contributed less than I expected.
3 - The other agents contributed slightly less than I expected.
4 - The other agents contributed about the same as I expected.
5 - The other agents contributed slightly more than I expected.
6 - The other agents contributed more than I expected.
7 - The other agents contributed a lot more than I expected.

6. *[if group was not successful in game 2 but they kept the earnings]* How satisfied were you with being able to keep the earnings of game 2, even if your group did not achieve the target?

1. Completely dissatisfied
2. Mostly dissatisfied
3. Somewhat dissatisfied
4. Neither satisfied or dissatisfied
5. Somewhat satisfied
6. Mostly satisfied
7. Completely satisfied

6. *[if group was not successful in game 2 and they didn't keep the earnings]* How satisfied were you with NOT being able to keep the earnings of game 2, after your group not achieving the target?

1. Completely dissatisfied
2. Mostly dissatisfied
3. Somewhat dissatisfied
4. Neither satisfied or dissatisfied
5. Somewhat satisfied
6. Mostly satisfied
7. Completely satisfied

Part 3

1. How hard was it to set up the agent?

1. Very Difficult
2. Difficult
3. Neutral
4. Easy
5. Very easy

2. Did you understand how the agent works?

Yes
No
To a degree

2.1. *[If 2. != Yes]* What was it that you did not understand? Could you tell us in a sentence or two? (optional) *[answer: open text]*

3. Is there any extra option you would have liked to have when setting up your agent?

Yes
No
To a degree

3.1. *[If 3. != No]* Which ones? Please, elaborate below. (optional) *[answer: open text]*

4. Do you trust that the agent did what you instructed it to do?

Yes
No
To a degree

4.1. *[If 4. = Yes]* Why did you trust the agent? Please, elaborate briefly. (optional)
4.1. *[If 4. = To a degree]* Why did you not (fully) trust the agent? Please, elaborate briefly. (optional)
4.2. *[If 4. != Yes]* What would you need to trust the agent (more)? Please, elaborate briefly. (optional)

Part 4

1.1. Of the following, what would be your most preferred outcome?
The group achieving the target.
Getting the highest payoff of the group.
Everyone getting the same earnings.
I have no preference.
1.2. *[If 1.1. = The group achieving the target.]* And of the following, what would be your most preferred outcome?
Getting the highest payoff of the group.
Everyone getting the same earnings.
I have no preference.
1.2. *[If 1.1. = Getting the highest payoff of the group.]* And of the following, what would be your most preferred outcome?
The group achieving the target.
Everyone getting the same earnings.
I have no preference.
1.2. *[If 1.1. = Everyone getting the same earnings.]* And of the following, what would be your most preferred outcome?
The group achieving the target.
Getting the highest payoff of the group.
I have no preference.
2. Of the following, what do you think is the most fair outcome?
The group achieving the target.
Everyone getting the same earnings.
Everyone contributing the same amount.
3. Do you think the other group members agree with you on question 2. ?
Definitely no
No
Probably no
I am not sure
Probably yes
Yes
Definitely yes
4. *[If 3. is Probably no/No/Definitely no]* Since you think they would consider a different option as the most fair outcome: of the following, what do you think would be the most fair outcome for them?
The group achieving the target.
Everyone getting the same earnings.
Everyone contributing the same amount.

No-delegation. The following is the questionnaire shown at the end to all participants that took part in no-delegation experiments. This questionnaire is divided in 3 different parts. In the Part 1, the summary of the results of game 1 is shown again to the participants. In Part 2, the summary of both game 1 and game 2 are shown to the participants.

Part 1

1. How satisfied were you with the amount you earned in game 1?
   1. Completely dissatisfied
   2. Mostly dissatisfied
   3. Somewhat dissatisfied
   4. Neither satisfied or dissatisfied
   5. Somewhat satisfied
   6. Mostly satisfied
   7. Completely satisfied
2. *[if group was successful in game 1]* How satisfied were you with your group achieving the Public Account target of 80 ECoins in game 1?
   1. Completely dissatisfied
   2. Mostly dissatisfied
   3. Somewhat dissatisfied
   4. Neither satisfied or dissatisfied
   5. Somewhat satisfied
   6. Mostly satisfied
   7. Completely satisfied
2. *[if group was not successful in game 1]* How satisfied were you with your group NOT achieving the Public Account target of 80 ECoins in game 1?

1. Completely dissatisfied
2. Mostly dissatisfied
3. Somewhat dissatisfied
4. Neither satisfied or dissatisfied
5. Somewhat satisfied
6. Mostly satisfied
7. Completely satisfied

3. Which of the following best describe the expectations you had for the contributions of the other players in your group in game 1?

1. The other players contributed much less than I expected.
2. The other players contributed less than I expected.
3. The other players contributed slightly less than I expected.
4. The other players contributed about the same as I expected.
5. The other players contributed slightly more than I expected.
6. The other players contributed more than I expected.
7. The other players contributed a lot more than I expected.

4. *[if group was not successful in game 1 but they kept the earnings]* How satisfied were you with being able to keep the earnings of game 1, even if your group did not achieve the target?

1. Completely dissatisfied
2. Mostly dissatisfied
3. Somewhat dissatisfied
4. Neither satisfied or dissatisfied
5. Somewhat satisfied
6. Mostly satisfied
7. Completely satisfied

4. *[if group was not successful in game 1 and they didn't keep the earnings]* How satisfied were you with NOT being able to keep the earnings of game 1, after your group not achieving the target?

1. Completely dissatisfied
2. Mostly dissatisfied
3. Somewhat dissatisfied
4. Neither satisfied or dissatisfied
5. Somewhat satisfied
6. Mostly satisfied
7. Completely satisfied

Part 2

1. How satisfied were you with the amount you earned in game 2?

1. Completely dissatisfied
2. Mostly dissatisfied
3. Somewhat dissatisfied
4. Neither satisfied or dissatisfied
5. Somewhat satisfied
6. Mostly satisfied
7. Completely satisfied

2. *[if group was successful in game 2]* How satisfied were you with your group achieving the Public Account target of 80 ECoins in game 2?

1. Completely dissatisfied
2. Mostly dissatisfied
3. Somewhat dissatisfied
4. Neither satisfied or dissatisfied
5. Somewhat satisfied
6. Mostly satisfied
7. Completely satisfied

2. *[if group was not successful in game 2]* How satisfied were you with your group NOT achieving the Public Account target of 80 ECoins in game 2?

1. Completely dissatisfied
2. Mostly dissatisfied
3. Somewhat dissatisfied
4. Neither satisfied or dissatisfied
5. Somewhat satisfied
6. Mostly satisfied
7. Completely satisfied

3. Which of the following best describe the expectations you had for the contributions of the other players in your group in game 2?

1. The other players contributed much less than I expected.
2. The other players contributed less than I expected.
3. The other players contributed slightly less than I expected.
4. The other players contributed about the same as I expected.
5. The other players contributed slightly more than I expected.
6. The other players contributed more than I expected.
7. The other players contributed a lot more than I expected.

4. *[if group was not successful in game 2 but they kept the earnings]* How satisfied were you with being able to keep the earnings of game 2, even if your group did not achieve the target?

1. Completely dissatisfied
2. Mostly dissatisfied
3. Somewhat dissatisfied
4. Neither satisfied or dissatisfied
5. Somewhat satisfied
6. Mostly satisfied
7. Completely satisfied

4. *[if group was not successful in game 2 and they didn't keep the earnings]* How satisfied were you with NOT being able to keep the earnings of game 2, after your group not achieving the target?

1. Completely dissatisfied
2. Mostly dissatisfied
3. Somewhat dissatisfied
4. Neither satisfied or dissatisfied
5. Somewhat satisfied
6. Mostly satisfied
7. Completely satisfied

Part 3

1.1. Of the following, what would be your most preferred outcome?

The group achieving the target.
Getting the highest payoff of the group.
Everyone getting the same earnings.
I have no preference.

1.2. *[If 1.1. = The group achieving the target.]* And of the following, what would be your most preferred outcome?

Getting the highest payoff of the group.
Everyone getting the same earnings.
I have no preference.

1.2. *[If 1.1. = Getting the highest payoff of the group.]* And of the following, what would be your most preferred outcome?

The group achieving the target.
Everyone getting the same earnings.
I have no preference.

1.2. *[If 1.1. = Everyone getting the same earnings.]* And of the following, what would be your most preferred outcome?

The group achieving the target.
Getting the highest payoff of the group.
I have no preference.

2. Of the following, what do you think is the most fair outcome?

The group achieving the target.
Everyone getting the same earnings.
Everyone contributing the same amount.

3. Do you think the other group members agree with you on question 2. ?

Definitely no
No
Probably no
I am not sure
Probably yes
Yes
Definitely yes

4. *[If 3. is Probably no/No/Definitely no]* Since you think they would consider a different option as the most fair outcome: of the following, what do you think would be the most fair outcome for them?

The group achieving the target.
Everyone getting the same earnings.

Everyone contributing the same amount.

K-Means clustering

With Fig.2 of the Results section within the main manuscript the different individual behavioral profiles are shown after being clustered into 4 groups to facilitate further analysis. Here, we elaborate on the clustering technique and the assumptions used to arrive at such results.

We have chosen to work with the K-Means clustering technique because it relies on calculating distances between data points and possible centroids of the cluster in order to assign each data point to a cluster. Due to the geometric nature of the data points we use (a 10-element vector comprising all the actions taken by a participant in a game, which can also be interpreted as a 10-dimensional point in a space where each dimension belongs to the same range $[0, 4]$), it makes sense to use this distance-based method. On top of this, it runs fast for a sample size like ours $N_{total} = 920$.

With the goal of clustering the individual behaviors observed across treatments, it was important to decide whether or not to cluster the different treatment conditions together or not. In what concerns mixing delegation with no-delegation treatments, the choice was simple: since the goal is to identify what actually happened during the course of the game, rather than extracting the intuition or reasoning behind the participant's course of action, we have decided to cluster the round-by-round behavior of delegation together with the ones from no-delegation treatment (rather than focusing on clustering the settings in one case, and the round-by-round behavior in another, for example).

Another question was whether it made sense to cluster the behavioral profiles from different number of choices conditions together, a pertinent question since actually the participants in the 3 choices treatment condition were not able to use as many different options as the ones in the 5 choices treatment condition. For this matter, we follow the K-Means methodology for the 3 cases: considering only the data on the 3 action treatment, considering only the data on the 5 action treatment and considering all data together. First, we apply to each case the elbow method, indicative of how many clusters to group our data on (the only parameter needed to fine-tune the K-Means algorithm), for which the results are shown in Figs. S1, S2 and S3 respectively. In these figures it is evident that there is no doubt: within our data 4 main behavioral profiles can be identified irregardless of the number of actions permitted to the participants in their assigned treatment. It was still relevant to check whether the centroids of the 4 clusters found within each treatment matched with one another, or if the 4 behavioral profiles all differed from one another. To this end, we present the cluster centers in Table S18 where similar centroids are clearly found to identify each cluster even when comparing between treatments.

For the implementation of this clustering technique we have relied on the python package *sklearn.cluster.KMeans* (https://scikit-learn.org/stable/modules/generated/sklearn.cluster.KMeans.html). Specifically we have first defined the KMeans object by specifying the number of clusters $k = 4$ and the random state as 42 (relevant for reproduceability purposes, since KMeans relies on a random initialization of the cluster centroids). After defining the KMeans model, we have made use of the method *kmeans.fit_predict()* where the only input we have specified was the data to be clustered. This method then returned us the different cluster labels for each data point. Calling *kmeans.cluster_centers_* and rounding the result to two decimals, we were able to present the values in Table S18.

Accuracy vs Precision

As it is referred in the main text of the manuscript, one of the main differences encountered between participants in the delegation vs no-delegation treatment is the lack of precision that the groups in the first treatment present while trying to aim for the collective target of 80 (see Fig. S4). This interplay has already been addressed in different aspects of human behavior, namely in (6) where instead of accuracy vs precision, the authors choose the words "bias vs noise" and focus the book on the effects of noise in human decision-making related to business applications such as insurance brokerage. One way to easily illustrate the difference between accuracy and precision is to make use of a shooting at target task. Indeed, this is the example chosen by the authors of (6), as well as any educator trying to explain these concepts (just Google precision vs accuracy and you will find similar diagrams). Fig. S5 shows our own version of the famous (though unoriginal) diagram, where we present how 4 different group measurements would look like if the aim of the task was to shoot for a target. On top right, we see that the group exhibits both precision and accuracy (all shots happen on the bullseye - similar to what we find for the successful no-delegation groups in Fig. S4), top left shows lack of accuracy but precision (all shots are close to each other but away from the bullseye), bottom left shows lack of precision but accuracy (although no shot is on the bullseye, they are all around it - similar to what we find for the delegation groups in Fig. S4) and bottom right shows no accuracy or precision (shots are away from each other and their average is not close to the target).

Table S1. **4 different treatment conditions that arise from the 2×2 factorial design and respective sample sizes. In the process of data cleaning, only complete groups were kept for data analysis, so that for every group there are $4\times$individuals in every case. The total sample size is of 115 groups and 460 participants. The asymmetry observed between the 3 and the 5 choice treatment conditions is due to budgetary constraints that did not allow us to balance our sample, even though future work required the collection of more data points for further analysis on individual behavior in the 5 choice delegation condition.**

	Delegation treatment?	
#choices in the action space	Yes	No
3	27	22
5	44	22

Table S2. Contingency table for the frequency of successful groups in Game 1 per delegation treatment. A Fisher's exact test with alternative "less" (we hypothesize that delegation has lower frequency of success) yields a p-value of 0.001. We consider the total sample size of $n = 66$ groups, with 44 groups in the delegation and 22 in the no-delegation treatment.

	Successful?	
Condition	Yes	No
No-delegation with 5 actions	20	2
Delegation with 5 actions	22	22

Table S3. Contingency table for the frequency of successful groups in Game 2 per delegation treatment. A Fisher's exact test with alternative "less" (we hypothesize that delegation has lower frequency of success) yields a p-value of 0.001. We consider the total sample size of $n = 66$ groups, with 44 groups in the delegation and 22 in the no-delegation treatment.

	Successful?	
Condition	Yes	No
No-delegation with 5 actions	20	2
Delegation with 5 actions	23	21

Table S4. Contingency table for the frequency of behavioral profiles identified with cluster high-contributors per delegation treatment. A Fisher's exact test with alternative "greater" (we hypothesize that delegation has higher frequency of high-contributors behaviors) yields a p-value of $\ll 0.001$. Because it is a variable that pertains to the individual and is agnostic with regards to Game 1 or Game 2 for this analysis, we consider a total sample size of $N_{total} = 920$, with 568 samples belonging to the delegation and 352 to the no-delegation treatment.

	Is high-contributors?	
Delegation	Yes	No
Yes	122	446
No	18	334

Table S5. Contingency table for the frequency of behavioral profiles identified with cluster early-contributors per delegation treatment. A Fisher's exact test with alternative "less" (we hypothesize that delegation has lower frequency of early-contributors behaviors) yields a p-value of $\ll 0.001$. Because it is a variable that pertains to the individual and is agnostic with regards to Game 1 or Game 2 for this analysis, we consider a total sample size of $N_{total} = 920$, with 568 samples belonging to the delegation and 352 to the no-delegation treatment.

	Is early-contributors?	
Delegation	Yes	No
Yes	28	540
No	126	226

Table S6. Contingency table for the frequency of behavioral profiles identified with cluster low-contributors per number of choices treatment within the no-delegation condition. A Fisher's exact test with alternative "greater" (we hypothesize that no-delegation with 3 actions has higher frequency of low-contributors behaviors than no-delegation with 5 actions) yields a p-value of $\ll 0.001$. Because it is a variable that pertains to the individual and is agnostic with regards to Game 1 or Game 2 for this analysis, although only for the no-delegation condition, we consider a total sample size of $N_{no-d} = 352$, with 176 samples in both the 3 actions and the 5 actions treatment.

	Is low-contributors?	
Number of choices	Yes	No
3 actions	41	135
5 actions	14	162

Table S7. Contingency table for comparing the frequency of successful groups between previously successful and previously failed groups in the no-delegation treatment. A Fisher's exact test with alternative "greater" (we hypothesize that individuals within groups that were previously successful will be more successful in the second game) yields a p-value$= 0.024$.

	Previously successful?	
Successful in Game 2?	No	Yes
No	4	5
Yes	3	32

Table S8. Contingency table for comparing the frequency of cluster changes per delegation treatment. A Fisher's exact test with alternative "greater" (we hypothesize that individuals within the delegation treatment will change behaviors more frequently) yields a p-value$= 0.013$.

	Changed clusters?	
Delegation	Yes	No
Yes	141	143
No	68	108

Table S9. Summary of Welch's t-test performed on the results of Fig. 3 in the main text. The columns indicate for which number of choices condition the test is being performed (#choices), which Strategy is at focus (1 or 2, following notation in Figs. 3C and D of the main text), and which parameters are being compared between clusters (given by "others previous round" following also the notation in Figs. 3C and D of the main text). Finally the hypothesis is specified: we indicate which cluster is expected to be greater than another, or if they are even different, for example we hypothesize that high-contributors contribute more than fair-contributors and we indicate this with "high>fair" in this column; and we indicate the t-statistic and the p-values referring to the Welch's t-test applied to these hypothesis in the last columns.

#choices	Strategy	others previous round	Hypothesis	t-statistic	p-value
3	1	2	early≠high	-0.44	0.67
3	1	2	early>fair	3.21	0.004
3	1	2	high>fair	8.56	$\ll 0.001$
3	1	2	fair>low	7.14	$\ll 0.001$
3	2	2	high>fair	6.08	$\ll 0.001$
3	2	2	high>early	4.00	$\ll 0.001$
3	2	2	fair>low	2.01	0.024
3	2	2	fair>early	1.50	0.08
5	1	1	early≠high	-0.53	0.60
5	1	2	early≠high	0.19	0.85
5	1	3	early≠high	1.02	0.32
5	1	1	early>fair	2.43	0.013
5	1	2	early>fair	4.47	$\ll 0.001$
5	1	3	early>fair	3.01	0.004
5	1	1	high>fair	4.91	$\ll 0.001$
5	1	2	high>fair	7.10	$\ll 0.001$
5	1	3	high>fair	3.57	$\ll 0.001$
5	1	1	fair>low	4.74	$\ll 0.001$
5	1	2	fair>low	8.34	$\ll 0.001$
5	1	3	fair>low	6.29	$\ll 0.001$
5	2	1	high>fair	3.64	$\ll 0.001$
5	2	2	high>fair	4.00	$\ll 0.001$
5	2	3	high>fair	3.38	$\ll 0.001$
5	2	1	high>early	2.92	0.004
5	2	2	high>early	8.45	$\ll 0.001$
5	2	3	high>early	5.30	$\ll 0.001$
5	2	1	fair>low	3.38	$\ll 0.001$
5	2	2	fair>low	4.07	$\ll 0.001$
5	2	3	fair>low	3.33	$\ll 0.001$
5	2	1	fair>early	1.35	0.097
5	2	2	fair>early	7.53	$\ll 0.001$
5	2	3	fair>early	3.52	$\ll 0.001$

Table S10. ANOVA summary table with the results pertaining to the effect of (no-)delegation and the number of choices in the action space on the Public Account values in Game 1. Sample size is $n = 115$ groups.

Source	SS	DF	MS	F	p-unc	np2
delegation	715.167	1.000	715.167	2.325	0.130	0.021
choice	730.269	1.000	730.269	2.374	0.126	0.021
delegation * choice	2846.470	1.000	2846.470	9.252	0.003	0.077
Residual	34148.917	111.000	307.648	nan	nan	nan

Table S11. ANOVA summary table with the results pertaining to the effect of (no-)delegation and the number of choices in the action space on the Public Account values in Game 2. Sample size is $n = 115$ groups.

Source	SS	DF	MS	F	p-unc	np2
delegation	963.755	1.000	963.755	2.815	0.096	0.025
choice	794.125	1.000	794.125	2.319	0.131	0.020
delegation * choice	1844.320	1.000	1844.320	5.386	0.022	0.046
Residual	38006.998	111.000	342.405	nan	nan	nan

Table S12. ANOVA summary table with the results pertaining to the effect of (no-)delegation and the number of choices in the action space on the in-group Private Account variance values in Game 1. Sample size is $n = 115$ groups.

Source	SS	DF	MS	F	p-unc	np2
delegation	50316.339	1.000	50316.339	18.450	0.000	0.143
choice	8687.301	1.000	8687.301	3.186	0.077	0.028
delegation * choice	5426.283	1.000	5426.283	1.990	0.161	0.018
Residual	302708.826	111.000	2727.107	nan	nan	nan

Table S13. ANOVA summary table with the results pertaining to the effect of (no-)delegation and the number of choices in the action space on the in-group Private Account variance values in Game 2. Sample size is $n = 115$ groups.

Source	SS	DF	MS	F	p-unc	np2
delegation	98320.362	1.000	98320.362	27.677	0.000	0.200
choice	6287.609	1.000	6287.609	1.770	0.186	0.016
delegation * choice	7838.749	1.000	7838.749	2.207	0.140	0.019
Residual	394316.273	111.000	3552.399	nan	nan	nan

Table S14. ANOVA summary table with the results pertaining to the effect of (no-)delegation and the number of choices in the action space on the Participation Frequency values in Game 1. Sample size is $n = 460$ individuals.

Source	SS	DF	MS	F	p-unc	np2
delegation	1.744	1.000	1.744	37.070	0.000	0.075
choice	1.569	1.000	1.569	33.364	0.000	0.068
delegation * choice	0.076	1.000	0.076	1.608	0.205	0.004
Residual	21.447	456.000	0.047	nan	nan	nan

Table S15. ANOVA summary table with the results pertaining to the effect of (no-)delegation and the number of choices in the action space on the Participation Frequency values in Game 2. Sample size is $n = 460$ individuals.

Source	SS	DF	MS	F	p-unc	np2
delegation	1.317	1.000	1.317	18.616	0.000	0.039
choice	2.481	1.000	2.481	35.077	0.000	0.071
delegation * choice	0.105	1.000	0.105	1.485	0.224	0.003
Residual	32.252	456.000	0.071	nan	nan	nan

Table S16. Agent configuration table as shown to the participants during the experimental treatment with 3 action choices. Within the main text we use the words "Strategy" instead of "Table". We decided to not use the same word in the text shown to the participants in order to not confuse or bias them. The participants were asked to fill in the values under "My agent will play in the next round:" through a free text numeric input.

**My agent starts to play:**	START
**Table 1**	
**When others played (on average in the current round):**	**My agent will play in the next round:**
**0**	TABLE 1 ROW 0
**2**	TABLE 1 ROW 2
**4**	TABLE 1 ROW 4
**My agent will switch to Table 2 when Public Account has:**	SWITCH
**Table 2**	
**When others played (on average in the current round):**	**My agent will play in the next round:**
**0**	TABLE 1 ROW 0
**2**	TABLE 1 ROW 2
**4**	TABLE 1 ROW 4

Table S17. Agent configuration table as shown to the participants during the experimental treatment with 5 action choices. Within the main text we use the words "Strategy" instead of "Table". We decided to not use the same word in the text shown to the participants in order to not confuse or bias them. The participants were asked to fill in the values under "My agent will play in the next round:" through a free text numeric input.

**My agent starts to play:**	START
**Table 1**	
**When others played (on average in the current round):**	**My agent will play in the next round:**
**0**	TABLE 1 ROW 0
**1**	TABLE 1 ROW 1
**2**	TABLE 1 ROW 2
**3**	TABLE 1 ROW 3
**4**	TABLE 1 ROW 4
**My agent will switch to Table 2 when Public Account has:**	SWITCH
**Table 2**	
**When others played (on average in the current round):**	**My agent will play in the next round:**
**0**	TABLE 1 ROW 0
**1**	TABLE 1 ROW 1
**2**	TABLE 1 ROW 2
**3**	TABLE 1 ROW 3
**4**	TABLE 1 ROW 4

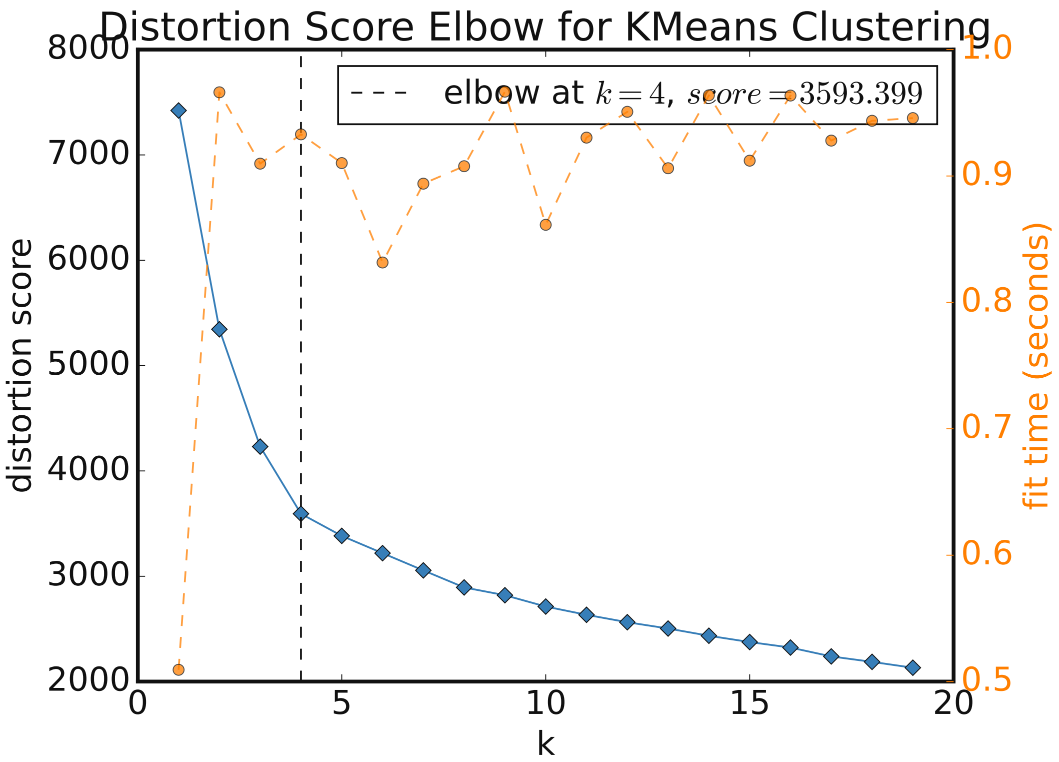

Fig. S1. Distortion Score Elbow for K-Means clustering on all the data where the number of choices available to the participants was 3 (action space being $S = \{0, 2, 4\}$). The data used encompasses the actions taken by the participants at each of the 10 rounds of the game. We cluster both games together, considering 2 entries for this dataset from each participant, one regarding Game 1 and the other Game 2, so that total sample size is $N_3 = 392$. This visualization was produced using the *KElbowVisualizer* visualizer within the python package *Yellowbrick* (see https://www.scikit-yb.org/en/latest/api/cluster/elbow.html).

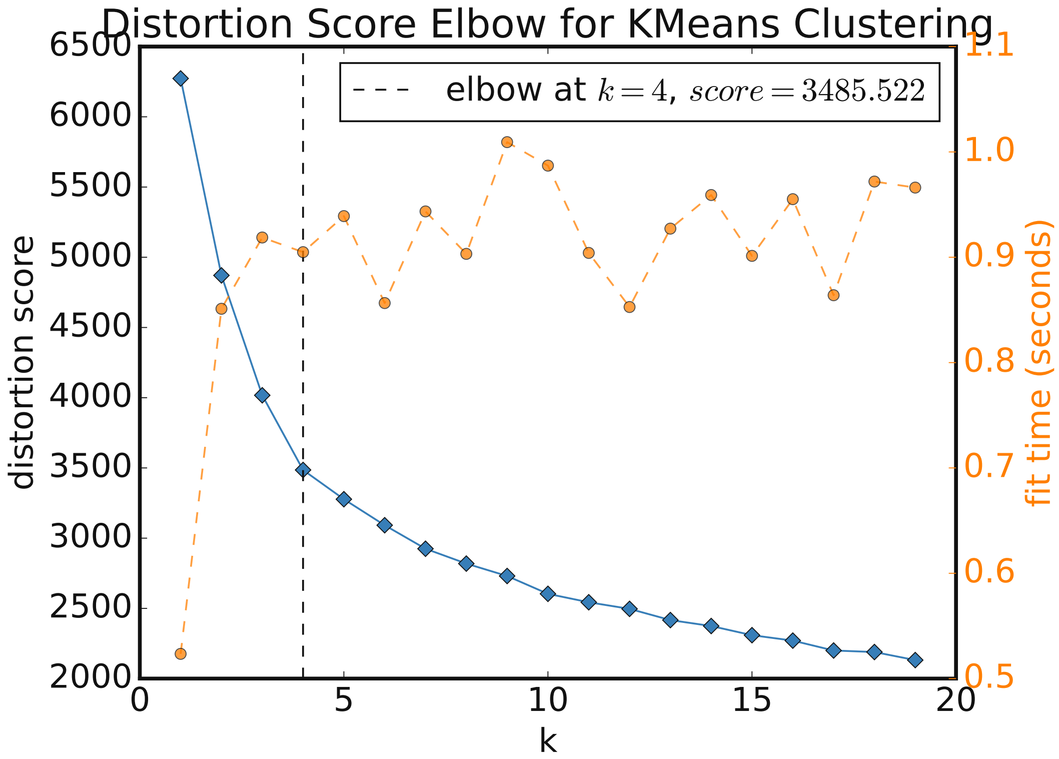

Fig. S2. Distortion Score Elbow for K-Means clustering on all the data where the number of choices available to the participants was 5 (action space being $S = \{0, 1, 2, 3, 4\}$). The data used encompasses the actions taken by the participants at each of the 10 rounds of the game. We cluster both games together, considering 2 entries for this dataset from each participant, one regarding Game 1 and the other Game 2, so that total sample size is $N_5 = 528$. This visualization was produced using the *KElbowVisualizer* visualizer within the python package *Yellowbrick* (see https://www.scikit-yb.org/en/latest/api/cluster/elbow.html).

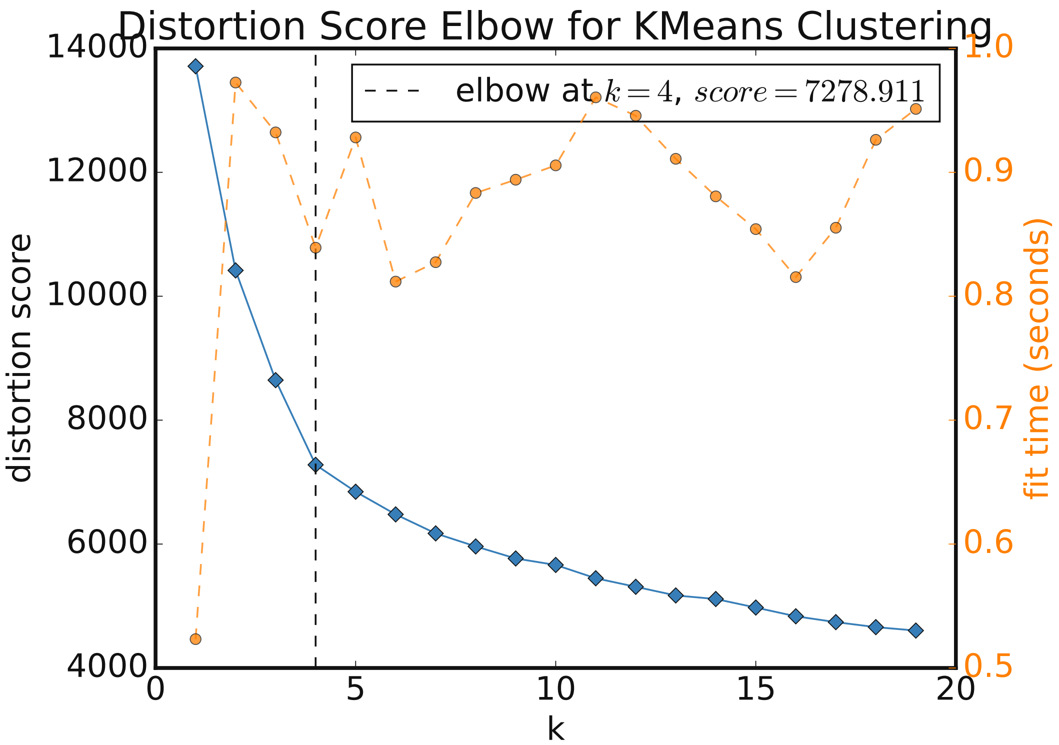

Fig. S3. Distortion Score Elbow for K-Means clustering on all the data. The data used encompasses the actions taken by the participants at each of the 10 rounds of the game. We cluster both games together, considering 2 entries for this dataset from each participant, one regarding Game 1 and the other Game 2, so that total sample size is $N_{total} = 920$. This visualization was produced using the *KElbowVisualizer* visualizer within the python package *Yellowbrick* (see https://www.scikit-yb.org/en/latest/api/cluster/elbow.html).

Table S18. Cluster centroids where each dimension is named as "Action" followed by the round number for each case: when we cluster only on 3 choices data, when we cluster only on the 5 choices data and when we cluster all data together. Clusters 3early-, 5early- and early- would therefore correspond to early-contributors as they start the game contributing high and they contribute low in the later rounds. Clusters 3high-, 5high- and high- correspond to high-contributors with a fixed high contribution behavior throughout the game. Clusters 3fair-, 5fair- and fair- correspond to fair-contributors. Clusters 3low-, 5low- and low- correspond to low-contributors with a fixed low contribution throughout the game.

Dataset	Cluster	Action 1	Action 2	Action 3	Action 4	Action 5	Action 6	Action 7	Action 8	Action 9	Action 10
	3early-	3.12	3.4	3.01	3.04	2.85	2.33	1.32	0.88	0.47	0.22
3 actions	3high-	2.16	3.34	3.67	3.61	3.61	3.77	3.7	3.61	3.57	3.31
	3fair-	2.15	2.09	2.11	2.02	1.97	1.99	2.11	2.1	2.01	1.86
	3low-	1.48	1.04	0.78	0.65	0.47	0.23	0.21	0.21	0.18	0.18
	5early-	3.1	3.48	3.33	3.14	2.96	2.12	1.65	0.96	0.4	0.3
5 actions	5high-	2.82	2.84	3.01	3.	3.01	3.	3.01	3.07	3.04	2.77
	5fair-	2.21	2.11	2.05	1.95	1.99	1.96	2.03	2.08	1.91	1.81
	5early-	1.9	1.3	0.97	0.95	0.95	0.94	1.01	0.91	0.8	0.91
	early-	3.12	3.45	3.23	3.16	2.92	2.23	1.58	0.97	0.47	0.3
All	high-	2.48	3.13	3.33	3.29	3.29	3.4	3.35	3.36	3.36	3.05
	fair-	2.22	2.06	2.05	1.96	1.97	1.93	2.04	2.07	1.92	1.82
	low-	1.67	1.19	0.85	0.75	0.71	0.64	0.55	0.51	0.46	0.51

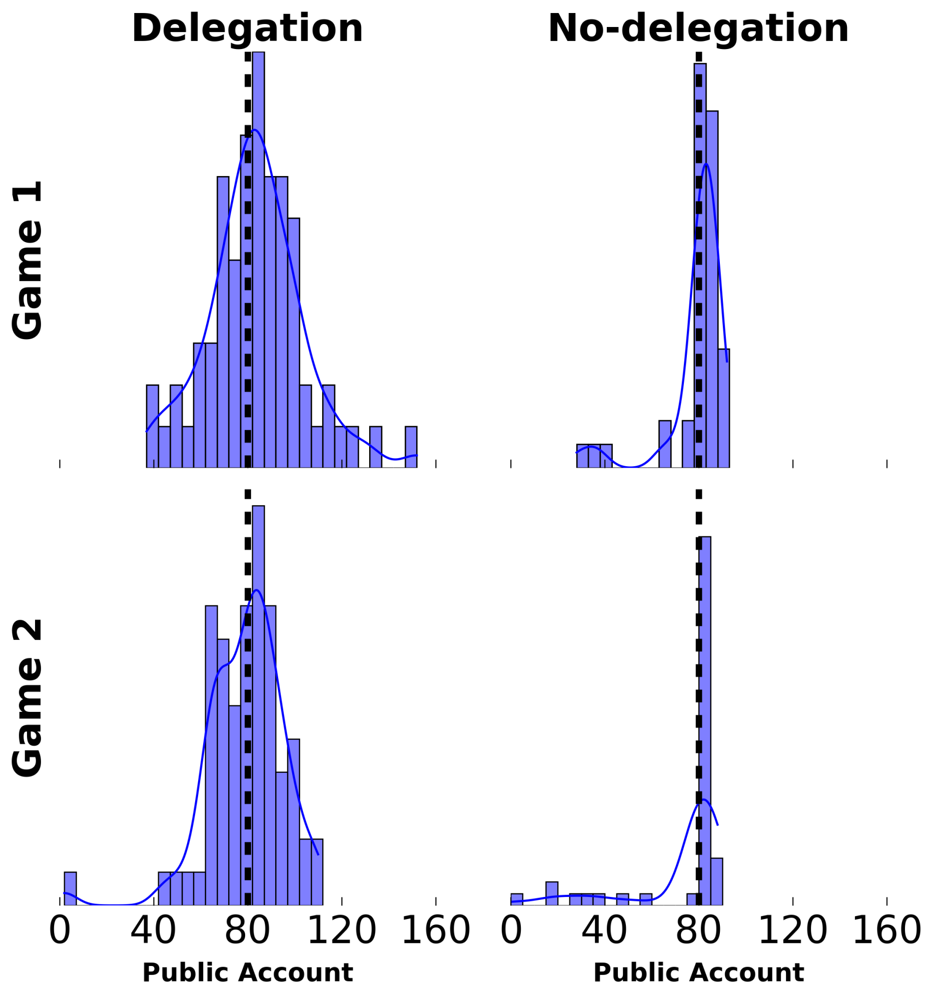

Fig. S4. Distribution of Public Account values between Game 1 (top) and Game 2 (bottom), for both treatment condition delegation (left) and no-delegation (right). Target 80 is marked with a dashed black line on top of each histogram. As shown in the picture, delegation Public Account values show accuracy around the intended target but with less precision than the no-delegation ones.

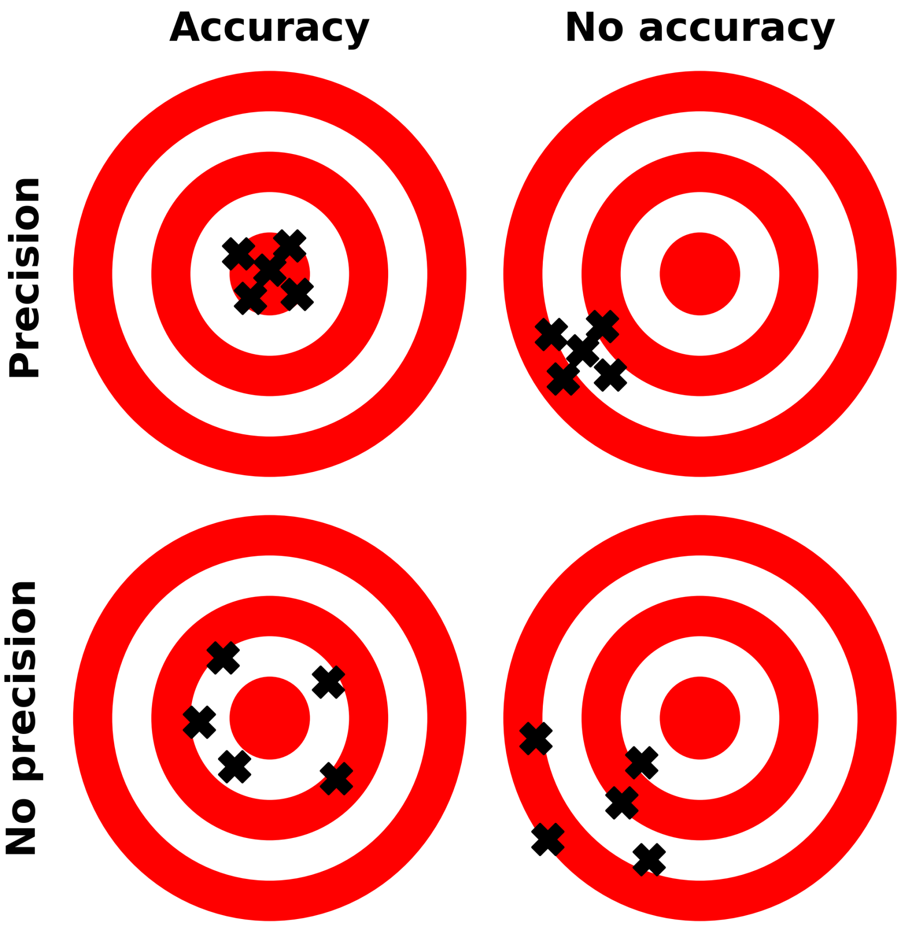

Fig. S5. Diagram to illustrate the definitions of precision and accuracy. Accuracy and precision are two measures of observational error. Accuracy is how close a given set of measurements (observations or readings) are to their true value, while precision is how close the measurements are to each other. In this work, we make use of these definitions to describe the distribution of Public Account values encountered in our data, with accuracy defining how close each Public Account was to the intended target of 80 and precision by how close the distribution of values were to each other within their treatment condition. A similar diagram can be found in (6) to explain the difference between bias and noise in human decision-making.

SI Dataset S1 (groupdf.csv)

This dataset includes all data that identifies a group (group number, treatment condition), their group results in each game (public account and whether it was successful or not), and the individual results aggregated per group (average, variance, maximum and minimum private account and action in each round for both games). The information pertains to $n = 115$ different groups. This was used to produce Figs. 1A-D, 2A-B and 4A in the main manuscript. Dataset is published in the platform Zenodo at https://doi.org/10.5281/zenodo.10805220 (7).

SI Dataset S2 (individual_data.csv)

This dataset includes all data that identifies an individual (player number, group number, treatment condition), their group results in each game (private and public account, whether or not their group was successful), individual actions throughout the game, agent configuration settings in case they belong to a delegation condition, participation frequency, change in success between game 1 and game 2, change in contributions between game 1 and game 2, change in cluster between game 1 and game 2, change in agent settings between game 1 and game 2 (only for delegation conditions), behavioral clusters in game 1 and in game 2. The information pertains to $N = 460$ different individuals. This was used to produce Figs. 1E-F, 2C-D and 4B-D in the main manuscript. Dataset is published in the platform Zenodo at https://doi.org/10.5281/zenodo.10805220 (7).

SI Dataset S3 (datalongStrats.csv)

This dataset is produced from Dataset S2 after a wide-to-long transformation with a focus on action per round. Therefore, it contains information that identifies each player's action (in each of the 10 rounds), the treatment condition and the game in which it is played. It contains 9200 data points, corresponding to the 10 round action in each of the 2 games played for all the 460 players recruited. This was used to produce Figs. 3A-B in the main manuscript. Dataset is published in the platform Zenodo at https://doi.org/10.5281/zenodo.10805220 (7).

SI Dataset S4 (datalongDel.csv)

This dataset is produced from Dataset S2 after a wide-to-long transformation with a focus on agent configuration settings. Therefore, it contains information that identifies each player's agent configuration setting (12 settings in the 5 action treatment and 8 settings in the 3 action treatment, for both games and without considering the setting $SWITCH$), the treatment condition and the game in which it is played. It contains 5384 data points, corresponding to the 11 settings considered in both games for the 176 participants of the 5 action delegation condition; and the 7 settings considered in both games for the 108 participants of the 3 action delegation condition. This was used to produce Figs. 3C-D in the main manuscript. Dataset is published in the platform Zenodo at https://doi.org/10.5281/zenodo.10805220 (7).

SI Dataset S5 (cluster_data.csv)

This is the dataset used to cluster behavioral data irregardless of in which game the actions were played. It contains individual data regarding actions per round and agent configuration settings, as well as to which treatment condition does this individual data belong to (number of actions and delegation). Therefore, it contains a total of 920 data points: information for each of the $N = 460$ individuals in both games. This data set is used to produce Figs. 3E-F of the main text. Dataset is published in the platform Zenodo at https://doi.org/10.5281/zenodo.10805220 (7).

References

1. R Selten, Die strategiemethode zur erforschung des eingeschränkt rationalen verhaltens im rahmen eines oligopolexperimentes in *Seminar für Mathemat. Wirtschaftsforschung u. Ökonometrie.* (1965).
2. J Brandts, G Charness, The strategy versus the direct-response method: a first survey of experimental comparisons. *Exp. Econ.* **14**, 375–398 (2011).
3. W Minozzi, J Woon, Direct response and the strategy method in an experimental cheap talk game. *J. Behav. Exp. Econ.* **85**, 101498 (2020).
4. U Fischbacher, S Gächter, Social preferences, beliefs, and the dynamics of free riding in public goods experiments. *Am. economic review* **100**, 541–56 (2010).
5. U Fischbacher, S Gächter, S Quercia, The behavioral validity of the strategy method in public good experiments. *J. Econ. Psychol.* **33**, 897–913 (2012).
6. D Kahneman, O Sibony, CR Sunstein, *Noise: a flaw in human judgment.* (Hachette UK), (2021).
7. I Terrucha, et al., Supporting Datasets and Notebook for the manuscript: "Humans program artificial delegates to accurately solve collective-risk dilemmas but lack precision" (2024).
